# Age-dependent relationships among diet, body condition, and *Echinococcus multilocularis* infection in urban coyotes

**DOI:** 10.1371/journal.pone.0290755

**Published:** 2023-08-30

**Authors:** Scott Sugden, Deanna K. Steckler, Dana Sanderson, Bill Abercrombie, Duncan Abercrombie, M. Alexis Seguin, Kyra Ford, Colleen Cassady St. Clair

**Affiliations:** 1 Department of Biological Sciences, University of Alberta, Edmonton, Alberta, Canada; 2 Department of Natural Resource Sciences, McGill University, Sainte-Anne-de-Bellevue, Quebec, Canada; 3 Department of Biological Sciences, MacEwan University, Edmonton, Alberta, Canada; 4 Animal Damage Control, Bushman Inc., Sherwood Park, Alberta, Canada; 5 IDEXX Laboratories, Inc., Westbrook, Maine, United States of America; Universitat Autonoma de Barcelona, SPAIN

## Abstract

Urban coyotes (*Canis latrans*) in North America increasingly exhibit a high prevalence of *Echinococcus multilocularis*, a cestode of recent and rising public health concern that uses rodents as intermediate hosts and canids as definitive hosts. However, little is known about the factors that drive the high urban prevalence of this parasite. We hypothesized that the diet of urban coyotes may contribute to their higher *E*. *multilocularis* infection prevalence via either (a) greater exposure to the parasite from increased rodent consumption or (b) increased susceptibility to infection due to the negative health effects of consuming anthropogenic food. We tested these hypotheses by comparing the presence and intensity of *E*. *multilocularis* infection to physiological data (age, sex, body condition, and spleen mass), short-term diet (stomach contents), and long-term diet (δ^13^C and δ^15^N stable isotopes) in 112 coyote carcasses collected for reasons other than this study from Edmonton, Alberta and the surrounding area. Overall, the best predictor of infection status in this population was young age, where the likelihood of infection decreased with age in rural coyotes but not urban ones. Neither short- nor long-term measures of diet could predict infection across our entire sample, but we found support for our initial hypotheses in young, urban coyotes: both rodent and anthropogenic food consumption effectively predicted *E*. *multilocularis* infection in this population. The effects of these predictors were more variable in rural coyotes and older coyotes. We suggest that limiting coyote access to areas in which anthropogenic food and rodent habitat overlap (e.g., compost piles or garbage sites) may effectively reduce the risk of infection, deposition, and transmission of this emerging zoonotic parasite in urban areas.

## Introduction

Generalist species thrive in urban environments due to their ability to exploit anthropogenic resources [1,2], but urban habitat use can also alter ecological relationships in ways that increase the transmission and spread of parasites. For example, the resources in urban environments, including available habitat and anthropogenic food subsidies, are often concentrated in space or time, causing hosts or vectors to aggregate [3–5]. In addition, nutrient-poor diets and chemical pollutants in urban areas can decrease host health and immune function, thereby increasing susceptibility to parasites [6]. Because urban areas harbor unique assemblages of species that may not coexist elsewhere, urban-dwelling hosts may also be exposed to new parasites, and, conversely, parasites may encounter novel hosts such as humans or pets [7,8]. Many of the parasites that occur in urban wildlife are zoonotic, such as sarcoptic mange [9,10], *Toxoplasmosis* [11], and the raccoon roundworm [12]. Increasing populations of urban generalists presents new public health risks because they necessarily increase human exposure to the zoonotic parasites of these species [6,13,14].

One such zoonotic parasite of increasing concern in Canada is the trophically transmitted tapeworm *Echinococcus multilocularis* [15]. This tapeworm uses rodents (e.g., voles and mice) and canids (e.g., foxes and coyotes) as intermediate and definitive hosts, respectively [16]. Humans may become infected by accidentally ingesting parasite eggs, which most commonly occurs either via transmission from pets that have consumed infected feces or rodents or from the consumption of unwashed produce contaminated with infected feces [17,18]. In humans, *E*. *multilocularis* infections can lead to alveolar echinococcosis, a potentially fatal zoonosis [19]. Multiple strains of *E*. *multilocularis* occur throughout the northern hemisphere [20], and while the historical North American strain was not particularly infectious to humans [21], the more virulent European strain has recently arrived in Canada, where it has become widespread among wolves, foxes, and coyotes [22,23]. The increasing prevalence of this virulent *E*. *multilocularis* strain raises significant human health concerns because some definitive hosts, especially coyotes, are common inhabitants of urban areas across the continent.

The majority of locally acquired human *E*. *multilocularis* infections have been reported from the province of Alberta [24], where the parasite appears to be highly prevalent among coyotes in urban areas. Indeed, several recent studies by us and other authors have reported that over half of Edmonton’s urban coyote population carries *E*. *multilocularis* [25–27], an infection prevalence 50% higher than that of coyotes in the surrounding area [27]. However, the reasons for this uniquely high prevalence of *E*. *multilocularis* in Edmonton’s coyote population remain unknown. One potential driver is age: juveniles are generally more susceptible to parasites than adults across a range of host and parasite taxa [28–30], and our previous urban study population was younger, on average, than rural coyotes [27]. Several studies have indeed found that *E*. *multilocularis* parasitism can be age-dependent [31–34]. However, another potentially important driver of urban infections is diet: Edmonton’s urban coyotes consume more anthropogenic food and rodents and less overall protein than their rural counterparts [27,35,36], which may alter the transmission dynamics of a trophically transmitted parasite like *E*. *multilocularis*.

The first diet-related mechanism that may contribute to the high prevalence of *E*. *multilocularis* in Edmonton coyotes is greater exposure to the parasite via increased consumption of infected rodents. Studies of urban foxes in Europe have shown that *E*. *multilocularis* infection prevalence parallels rodent availability [37,38]; other European studies have found that *E*. *multilocularis* is less prevalent in urban foxes than in semi-urban or rural populations [32,39,40] because urban areas provide less rodent habitat and, given the availability of anthropogenic food subsidies, urban predators are less dependent on rodents [41,42]. However, Edmonton is unlike European urban areas because the city contains an abundance of rodent habitat stemming from its large, contiguous river valley, low human density, and high percentage of undeveloped land [43]. Coyotes in Edmonton correspondingly appear to specialize on rodents [35,36], especially voles [44], which are common intermediate hosts for *E*. *multilocularis* [45]. Edmonton’s unique setting and the known dietary habits of its coyote population suggest that Edmonton coyotes may have unusually frequent exposure to *E*. *multilocularis*.

A second diet-related mechanism that could drive the prevalence of *E*. *multilocularis* in Edmonton coyotes is higher susceptibility to the parasite due to the negative health effects of consuming nutrient-poor anthropogenic food [27,36,46]. Consumption of anthropogenic food has been associated with increased parasite burdens in several wildlife species due to suppressed immune function [47], poor nutrition [48], and aggregation of infected hosts [46]. Our previous work showed that protein-poor diets can alter the coyote gut microbiome and reduce body condition [27], which might increase susceptibility to infection [5]. We have also shown that coyotes in Edmonton aggregate at large, unsecured compost piles, which can contain immune-suppressing mycotoxins [49]; coyote scats collected from these compost piles were 10 times more likely to contain tapeworm eggs than scats collected elsewhere in the city [49]. However, coyotes that deposited parasite-rich feces at these compost sites could have exhibited high infections either because of the immune-suppressing effects of consuming compost, as we hypothesize here, or because they were exploiting an abundance of rodents that were also attracted to compost, as we hypothesize above. Disentangling these two mechanisms requires a more detailed analysis of the dietary factors that drive *E*. *multilocularis* infections in urban coyotes, which can then inform targeted strategies for reducing infection rates and the likelihood of human exposure.

Here, we assessed which of these two proposed dietary mechanisms (i.e., greater exposure to infected prey or greater susceptibility to infection) best explains the uniquely high prevalence of *E*. *multilocularis* infections in Edmonton’s urban coyote population in the context of other factors (e.g., age, sex, and body condition) that can also moderate infection dynamics. We measured both the presence and intensity of *E*. *multilocularis* infections in the intestines of coyotes collected within and outside the City of Edmonton. To quantify both short- and long-term diet, we used stomach contents and carbon (δ^13^C) and nitrogen (δ^15^N) stable isotopes. Based on these measures, our first hypothesis (increased exposure) would be supported by increased rodent consumption (indicated via stomach contents or δ^15^N values) in infected coyotes, and our second hypothesis (greater susceptibility) would be supported by increased anthropogenic food consumption (indicated via stomach contents or δ^13^C values). Our results target key dietary and contextual factors underlying the prevalence of an emerging zoonotic parasite in Alberta and provide an important foundation for wildlife managers looking to mitigate the public health risks associated with *E*. *multilocularis* in urban wildlife.

## Materials and methods

### Study area

**Fig 1:**
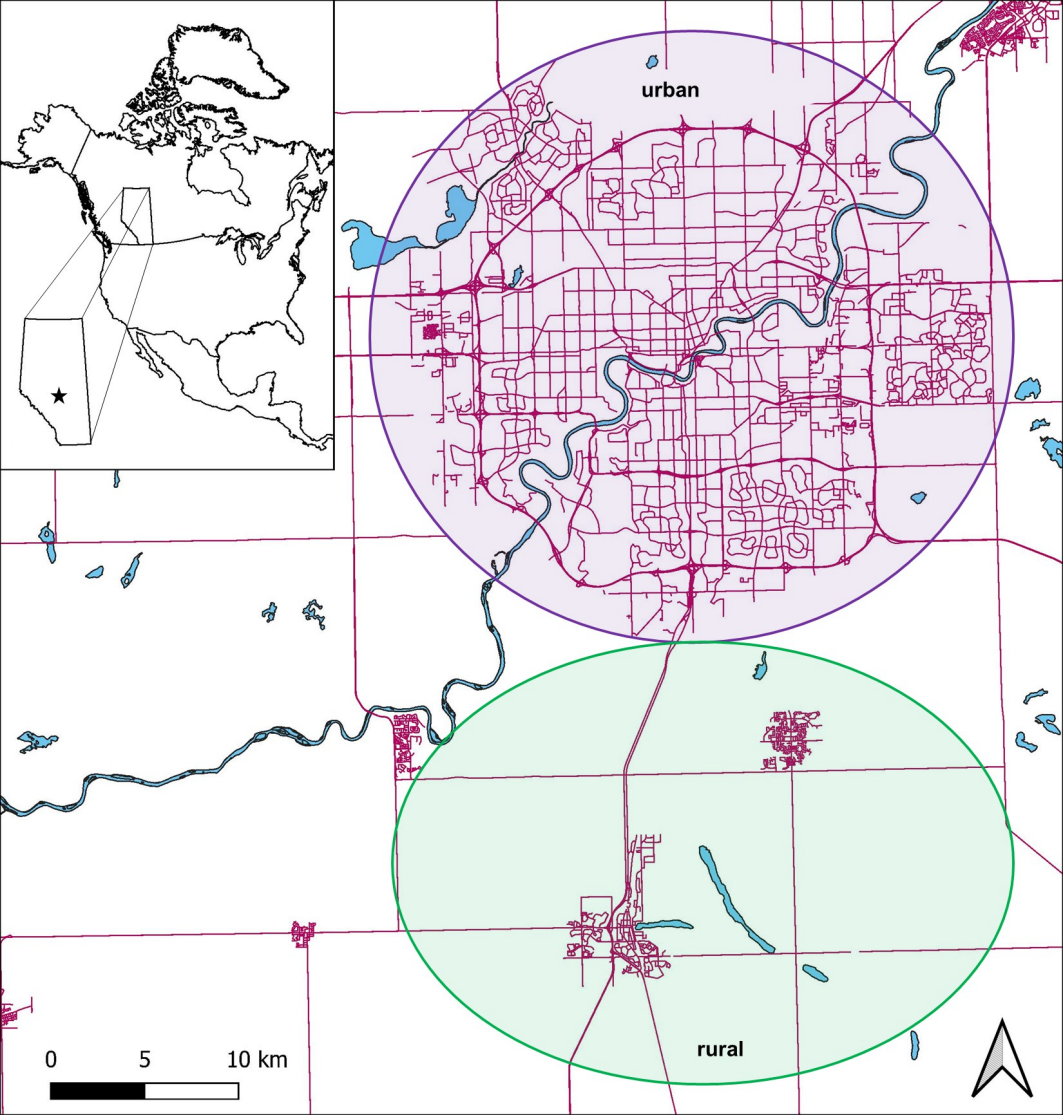
Map of the study area, with inset showing the location of Edmonton in central Alberta, Canada. Samples were collected from areas within (“urban”) and outside (“rural”) the city, as highlighted in the map. Roads (*purple*) and water bodies (*blue*) are shown for reference.

Coyote carcasses were collected between 2017 and 2020 from areas within (“urban”) and outside (“rural”) the City of Edmonton (**Fig 1; S1 Table** in **S1 Appendix**). Located in central Alberta, Edmonton has a population of approximately 1,000,000 residents and is bisected by the North Saskatchewan River, allowing for an extensive network of urban green spaces along the river valley and associated ravine network. These urban green spaces provide abundant habitat for coyotes as well as many of their small mammal prey species. Our rural study area was located south of Edmonton and encompassed several lakes and natural areas around the municipalities of Leduc and Beaumont. Climatically, the Edmonton region is characterized by a prairie climate with warm summers (high temperatures of 21°C to 23°C) and cold winters (average high temperatures of −3°C to −7°C).

### Sample collection and necropsy

All coyote carcasses were donated by outside sources following one of population management, roadkill, or conflict with humans. Carcasses were stored at -80°C for at least five days to neutralize the zoonotic risk of *E*. *multilocularis* [50] and then transferred to -20°C until necropsy. The carcasses used in this study include the carcasses used by Sugden et al. [27] as well as additional samples collected in subsequent years. We therefore followed the same necropsy procedures described by Sugden et al. [27]: we measured body size (mass, length, and girth), calculated the kidney fat index (KFI) as a metric for body condition [51], and divided spleen mass (in grams) by body mass (in kilograms) to obtain a coarse measure of immune system function [52]. We removed the small intestine for parasite analysis, the stomach for diet analysis, the outer hind claws for stable isotope analysis, and the lower jaw for age determination. Age was later determined by counting the cementum annuli of the lower canine teeth; teeth were fixed, decalcified, sectioned, stained, and visualized following published methods [53] as described by Sugden et al. [27].

### *E*. *multilocularis* detection and quantification

We used a combination of molecular and morphological methods to detect the presence and intensity of *E*. *multilocularis* infections in coyote intestines. Following the scraping-filtration-counting approach recommended by Gesy et al. [54], we first divided the small intestine into four equal lengths. Because *E*. *multilocularis* is less abundant in the anterior small intestine [55], we removed a ~0.5 g mucosal scraping from the second quarter for molecular analysis before rinsing and filtering each of the four intestinal quarters. To ensure that our molecular and morphological tests were spatially paired, we did not combine the filtrates from each intestinal quarter. Intestinal contents trapped on a 150 μm sieve were resuspended in distilled H_2_O (10–20 ml) and then fixed in 70% ethanol to a total volume of 60 ml. The sample from the second intestinal quarter was divided into four 15-ml aliquots for morphological parasite analysis.

For molecular analysis, we used a commercially available real-time polymerase chain reaction (qPCR) assay (*Echinococcus* RealPCR^TM^ Panel, IDEXX Laboratories, Inc.) to detect the presence of *E*. *multilocularis* in the small intestine. Mucosal scrapings were stored in sterile microtubes at -20°C and transported to IDEXX Laboratories, Inc. (West Sacramento, CA). Real-time PCR was performed using the LightCycler 480 system (Roche) with proprietary forward and reverse primers and hydrolysis probes. The *E*. *multilocularis* qPCR assay targets a ribosomal RNA sequence between the *cox1* and *cox2* genes. Real-time PCR was performed with seven quality controls as described in **S1 Appendix**.

For morphological parasite analyses, we counted worms in intestinal samples to determine the presence and intensity of *E*. *multilocularis* infections. We used a dissection microscope to observe one 15-ml aliquot of the contents from the second quarter of the intestine. All parasite scolexes were morphologically identified and counted [56]. For three samples that contained >1,000 worms in the first 1 ml of the aliquot, we estimated the total number of worms in the remaining sample by multiplying the count by 15 ml. All the scolexes we identified appeared to be *E*. *multilocularis*; however, because coyotes can also be infected by the related parasite *E*. *canadensis* and scolexes of the two species are difficult to visually discriminate [57], we only recorded worm counts for carcasses that tested positive for *E*. *multilocularis* via qPCR.

Conversely, due to the high sensitivity of the qPCR test, 22 samples tested positive for *E*. *multilocularis* but contained no visible worms in the second quarter of the intestine. We attributed this to either the presence of an exceptionally light infection (e.g., a single worm) or remnant DNA from a new or recently concluded infection. To ensure that our analyses targeted only animals with active infections (i.e., significant enough to be shedding eggs), we defined “biologically active” infections as those that were both qPCR-positive for *E*. *multilocularis* and had visual evidence of *Echinococcus* spp. scolexes. Based on these criteria, the 22 samples that were qPCR-positive but contained no visible worms were considered “uninfected” for all statistical analyses. A more detailed analysis of these samples is provided in **S2 Appendix**.

### Coyote diet analysis

We used measurements of short- and long-term diet to test our hypotheses that diet influences *E*. *multilocularis* infection patterns. Short-term diet was assessed using stomach contents, which provide evidence of the last meal(s) eaten prior to death. We removed all contents from the stomach and then measured the total stomach content volume (in ml) via water displacement [58]. Contents were then rinsed and sorted into nine mutually exclusive diet components: four prey items (ungulates, rodents, meso-mammals, and birds), two types of anthropogenic food (digestible and indigestible), vegetation, insects, and native fruit. We defined anthropogenic food as items that originated from human food production or waste processes; items of at least minimal nutritive value (e.g., apples, dog kibble, chicken bones, birdseed) were considered digestible, and non-nutritive trash items (e.g., food wrappers, plastic, leather scraps) were considered indigestible. The volume of each component was quantified by water displacement, as before. Diet components with non-zero volumes less than our minimum measurement sensitivity (0.5 ml) were assigned a volume of 0.1 ml.

To assess diet over a longer term, we measured carbon (δ^13^C) and nitrogen (δ^15^N) stable isotope signatures in claw samples, which represent diet over the past 6–8 months [59,60]. In general, higher δ^13^C values are associated with corn-based (i.e., anthropogenic) food consumption [61]. Higher δ^15^N values indicate increased protein (i.e., prey) consumption. While this measure does not specifically test our hypothesis about rodents, we assumed that higher δ^15^N values for urban coyotes would reflect increased rodent consumption because rodents are the most common prey item for coyotes in urban areas [36]. Claw samples were prepared for stable isotope analysis following previously described methods [27].

### Statistical analysis

All statistical analyses were conducted using R version 3.6.3 [62] and are described fully in **S1 Appendix**. Because worm counts were highly right-skewed (skewness 6.60, kurtosis 50.27), we truncated worm counts at 10,000, added a pseudo-count of 1, and then natural log-transformed the counts, producing a response variable that approximated a negative binomial distribution (**S1 Fig** in **S1 Appendix**). To reduce collinearity among our measures of body condition, we limited our assessment of body condition to (*i*) axis scores on the first principal component of a principal components analysis (PCA) performed on mass, body size, girth, and KFI (**S2 Fig, S2 Table** in **S1 Appendix**), and (*ii*) spleen mass (normalized by body mass), which was retained separately because it was not correlated with the other measures (**S2 Fig** in **S1 Appendix**). Insects were excluded from further analysis due to their low occurrence rate in our sample (n = 7 individuals), and we measured diet diversity by calculating the Shannon diversity index for each coyote’s stomach contents. Finally, we distinguished “juvenile” from “adult” coyotes based on whether they were younger or older, respectively, than the median age of our sample. This distinction was only used for the convenience of data visualization; numerical ages were used for all statistical analysis.

We then used univariate regressions to test for broad differences in infection status (presence/absence of a biologically active infection) and intensity (natural log-transformed worm counts) as a function of location, age, sex, body condition, stomach contents, diet diversity, and stable isotope values. Infection status and intensity were modeled with logistic and negative binomial regressions, respectively. For each response/predictor pair, we assessed the significance of their univariate relationship by comparing each regression to the corresponding null model using a likelihood ratio test, with significance defined at p < 0.05. All predictors were scaled to mean zero and unit variance prior to analysis, and we weighted urban samples proportionally to their abundance to account for their smaller population size in our sample. Results from the univariate regression models were further verified using chi-squared tests, Wilcox rank-sum tests, and Spearman’s rank correlations, as appropriate.

Because coyote diet and infection status have been shown to vary with both location and age in this sample [27,35] and elsewhere [31–33,63], we also tested interaction effects to determine whether the effect of a variable differed between urban and rural areas or across different ages, as described in **S1 Appendix**. In brief, we ran three additional models for each response/predictor pair, allowing each predictor to interact with (*i*) age, (*ii*) location, and (*iii*) the interaction between age and location. We determined the significance of each interaction using a likelihood ratio test, as before, where each model was compared to the model in which the focal predictor did not appear.

To identify the overall best dietary predictors of infection status and intensity, we extended our interaction models by allowing multiple measures of diet to appear in the same model. For these models, we chose only the dietary predictors that explicitly tested our hypotheses about rodent and/or anthropogenic food consumption. Given the exploratory nature of our questions, we chose to evaluate all model subsets using the R package *MuMIn* [64] and averaged predictor coefficients across all models with a delta Akaike information criterion (ΔAIC_C_) < 2. Before averaging, model coefficients were standardized by their partial standard deviation and adjusted based on model weight [65]. We quantified the predictive accuracy of the final models using Cohen’s kappa (for the logistic regression) and root mean-squared error (for the negative binomial regression) calculated from *k-*fold cross-validation with *k* = 5.

We lastly used ordination approaches to assess whether infection status or intensity were better predicted by a cluster of diet components than by individual diet items. For these analyses, we excluded ten coyotes with empty stomachs, converted diet component volumes to relative abundances, and then ordinated the stomach content data using the Bray-Curtis distance. Differences between diet composition and each of infection prevalence and intensity were assessed using permutational multivariate analyses of variance (PERMANOVAs) that also included age and location as predictors. All ordination analyses were implemented using the R package *vegan* [66].

## Results

We obtained 112 coyote carcasses from population control programs in rural areas (n = 66), roadkill in the City of Edmonton (n = 35), and lethal management due to conflict with humans (urban: n = 6; rural = 5) (**S1 Table** in **S1 Appendix:**), for a total of n = 71 rural and n = 41 urban samples. Coyotes ranged in age from 0.26 to 11.43 years (mean: 2.47±2.32, median: 1.78), with a similar age distribution in both urban and rural areas (**S3 Fig** in **S1 Appendix**). Rodents and anthropogenic food were each present in approximately 50% of stomachs, with voles being the most common rodent prey item (**S3 Table** in **S1 Appendix**). As in previous studies of Edmonton coyotes [27,35,46], stomach contents and stable isotope values indicated that urban coyotes consumed significantly more digestible anthropogenic food and smaller prey items (e.g., rodents) than rural coyotes (**S3-S5 Tables** in **S1 Appendix**). Conversely, rural coyotes consumed significantly more total prey, including meso-mammals and ungulates, as well as significantly more indigestible anthropogenic food (**S3-S5 Tables** in **S1 Appendix**). In both urban and rural areas, juvenile coyotes consumed a more diverse variety of smaller prey items, whereas older coyotes consumed more ungulates (**S3-S5 Tables** in **S1 Appendix**).

### *E*. *multilocularis* infection status and intensity

**Fig 2:**
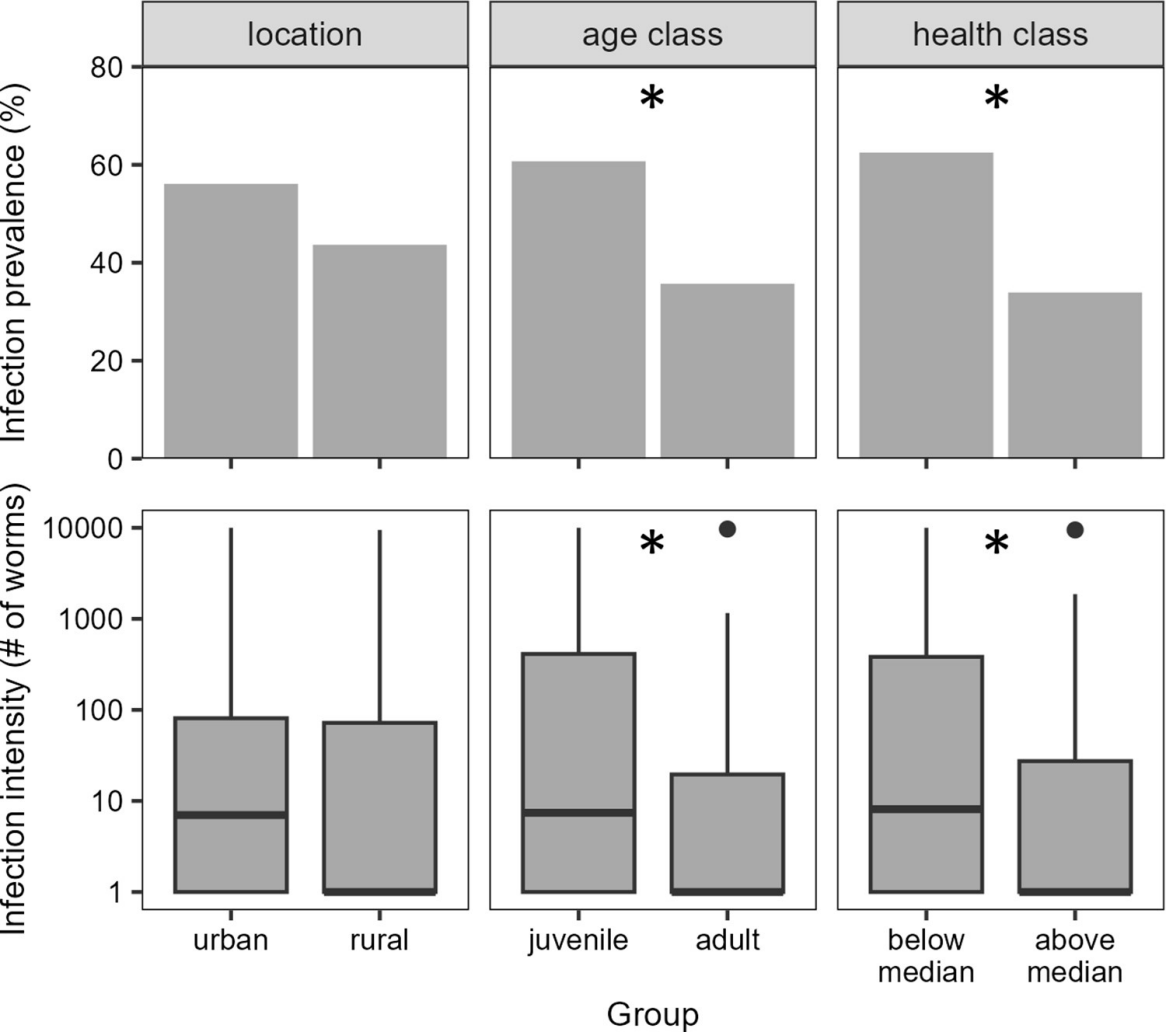
*E*. *multilocularis* prevalence and intensity compared between sampling locations (urban vs. rural, *left column*), age classes (juvenile vs. adult, *center column*), and health classes (above or below the median health score, *right column*). Prevalence is represented as the percentage of the sampled population, and infection intensity is represented as worm counts with a log-transformed y-axis. Juvenile and adult coyotes were distinguished as being younger or older, respectively, than the median age of our sample (1.78 yr). Significant differences (p < 0.05 in regression models; see **Methods** and **Table 1**) are represented with asterisks (*).

We detected biologically active *E*. *multilocularis* infections in 48.2% of the coyotes we examined, though 69.9% tested positive via qPCR (see **S2 Appendix**). The mean parasite load (± SD) was 1,351 ± 6,391 worms (range: 0–55,000); three urban individuals each carried >10,000 worms and accounted for 72% of all *E*. *multilocularis* scolexes we counted. Infection prevalence and intensity varied with both location and age: urban coyotes had a 28% higher infection prevalence than rural coyotes and carried an average of eight times more worms (**Fig 2**), though neither difference was significant in univariate regression models (**Table 1**). Infection prevalence and intensity were also significantly higher in younger coyotes and coyotes in poorer condition (**Fig 2**; **Table 1**), though we note that body condition was closely correlated with age (**S2 Fig** in **S1 Appendix**). We found no significant sex-based differences in either measure of infection (**Table 1**), and no single measure of diet—stomach content volumes, diet diversity, or stable isotope values—was able to predict infection status or intensity across our entire sample (**Table 1; S1 Table** in **S3 Appendix**). Similarly, neither infection status nor intensity were associated with multivariate representations of stomach contents in ordination analyses (**S1 Fig, S2 Table** in **S3 Appendix**).

**Table 1:** Results from likelihood ratio tests assessing relationships between contextual and dietary variables and each of infection status and intensity.

			infection status					infection intensity				
	infected	uninfected	univariate		interactions			univariate		interactions		
Variable	mean (sd)	mean (sd)	χ^2^	p	best model	χ^2^	p	χ^2^	p	best model	χ^2^	p
Context												
location	-	-	2.37	0.124	x age	13.66	**0.001**	1.28	0.257	x age	10.73	**0.005**
age	1.84 (1.90)	3.06 (2.53)	5.25	**0.022**	x location	16.54	**0.000**	5.41	**0.020**	x location	14.86	**0.001**
sex	-	-	0.17	0.681	-	-	-	0.05	0.830	-	-	-
body condition	-0.10 (0.43)	0.09 (0.43)	6.68	**0.010**	-	-	-	5.69	**0.017**	-	-	-
KFI	0.43 (0.30)	0.49 (0.30)	1.30	0.253	x age	6.94	**0.031**	1.37	0.242	x age	11.23	**0.004**
spleen size	1.98 (0.70)	1.99 (0.85)	0.08	0.782	x age x location	10.37	**0.035**	1.63	0.202	x age x location	16.98	**0.002**
Stomach contents												
total food volume	171.4 (374.2)	196.1 (290.0)	0.34	0.560	-	-	-	0.00	0.992	-	-	-
diet diversity	0.40 (0.38)	0.37 (0.37)	0.05	0.831	-	-	-	0.72	0.397	-	-	-
anthropogenic food	27.35 (68.38)	31.99 (70.54)	0.02	0.893	-	-	-	0.43	0.514	x age x location	13.35	**0.010**
digestible	18.95 (64.27)	30.2 (70.4)	0.35	0.555	x age x location	16.54	**0.002**	0.56	0.456	x age x location	17.32	**0.002**
indigestible	8.39 (26.68)	3 (13.22)	1.73	0.189	-	-	-	0.00	0.990	x age x location	11.33	**0.023**
prey items	141.2 (376.7)	158.1 (287.7)	0.22	0.635	-	-	-	0.03	0.867	-	-	-
rodent	23.68 (64.1)	26.5 (58.88)	0.14	0.704	x age x location	15.37	**0.004**	0.02	0.898	x age x location	18.70	**0.001**
meso-mammal	25.7 (106.4)	21.06 (59.46)	0.03	0.867	x age x location	10.15	**0.038**	0.17	0.679	-	-	-
ungulate	86.0 (360.9)	108.7 (288.6)	0.26	0.612	-	-	-	0.00	0.985	-	-	-
bird	5.73 (38.08)	1.77 (9.53)	0.47	0.492	-	-	-	0.32	0.574	-	-	-
vegetation	1.34 (2.72)	1.37 (3.25)	0.03	0.868	-	-	-	1.03	0.311	-	-	-
fruit	0.52 (2.45)	1.11 (7.89)	0.13	0.720	-	-	-	0.02	0.880	-	-	-
Stable isotopes												
d13C	-22.33 (1.32)	-22.39 (0.97)	0.65	0.421	-	-	-	0.93	0.336	x age	8.97	**0.011**
d15N	8.7 (0.93)	8.71 (0.9)	0.05	0.820	-	-	-	0.33	0.565	-	-	-

Mean values (±SD) for each predictor are shown for infected and uninfected coyotes. Infection status was modeled with logistic regressions, and infection intensity was modeled with negative binomial regressions. Significant univariate associations were assessed by comparing each response-predictor model to its corresponding null model (see **Methods**). We additionally tested whether the effect of each predictor varied with location or age by allowing each predictor to interact with (*i*) location, (*ii*) age, and (*iii*) the two-way interaction between location and age. The best interactions (p < 0.1) are shown for each predictor; missing values indicate that the predictor did not significantly improve the location, age, or location x age models.

**Fig 3:**
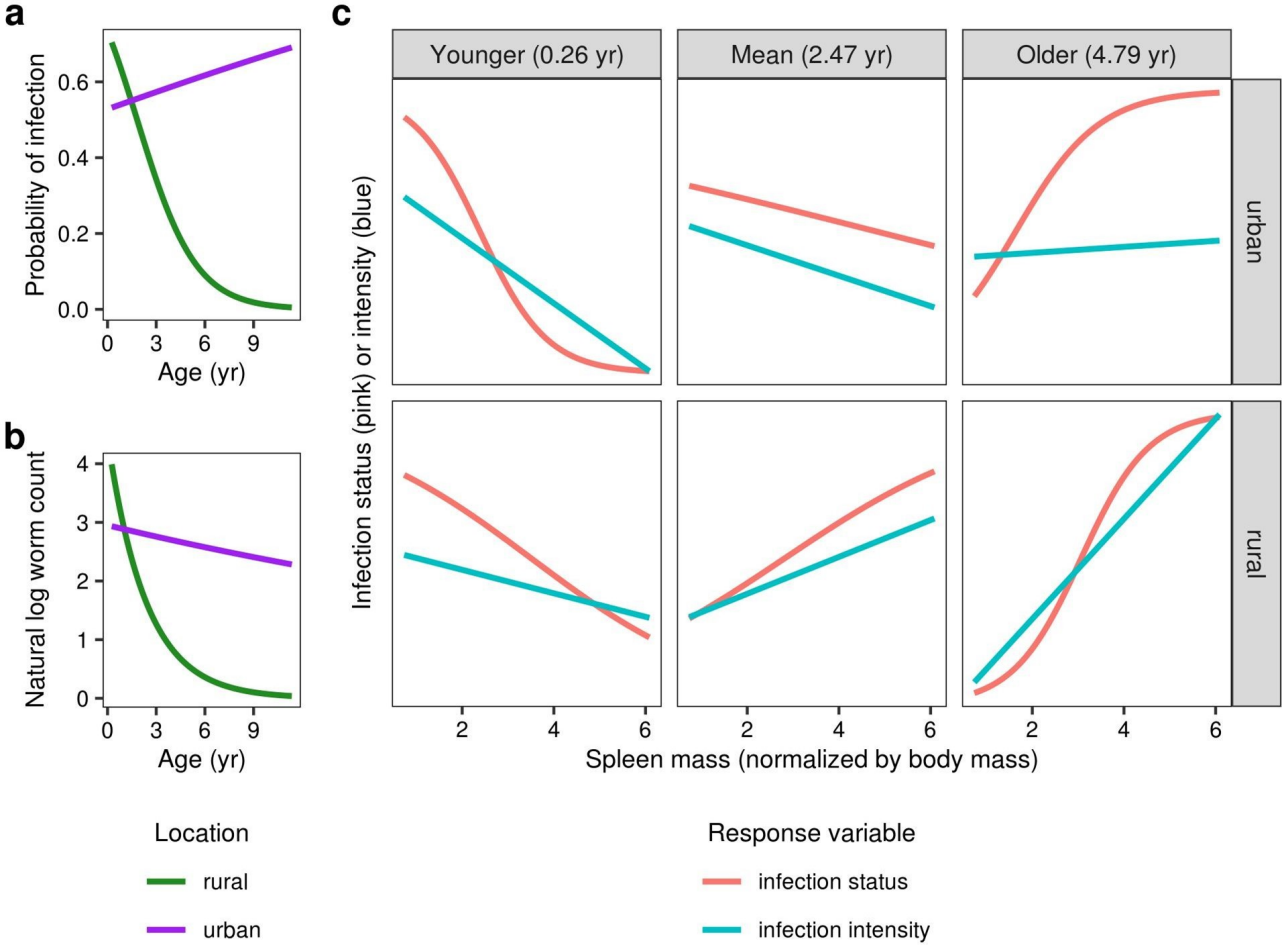
Interactions between age, location, and spleen mass (normalized by body mass, g kg^-1^) as predictors of *E*. *multilocularis* infection status and intensity. Infection status (**a**) and intensity (**b**) both declined significantly with age in rural coyotes but less so in urban coyotes. (**c**) Spleen mass exhibited a significant three-way interaction with age and location. The figure shows predicted relationships between spleen size and infection for urban and rural coyotes at three different ages (the mean age and ±1 standard deviation). Infection status (probability of infection) and intensity (natural log-transformed worm counts) were both adjusted to appear on the same y-axis.

A significant two-way interaction between location and age suggested that infection status and intensity declined with age in rural coyotes but not urban coyotes (**Fig 3A and 3B; Table 1**). We excluded our composite measure of body condition from our interaction analyses because of its correlation with age; we instead assessed spleen size alone, which did not exhibit this correlation. The relationship between spleen size and each of infection status and intensity exhibited a significant three-way interaction with location and age (**Table 1**). Specifically, young animals (< 1 yr) with larger spleens were less likely to be infected and carried fewer worms, but older animals with larger spleens were more likely to be infected and carried more worms (**Fig 3C; Table 1**). This reversing trend with age was consistent between urban and rural areas, though the age at which the relationship reversed was higher in urban coyotes (**Fig 3C**).

### Diet and infection status

**Fig 4:**
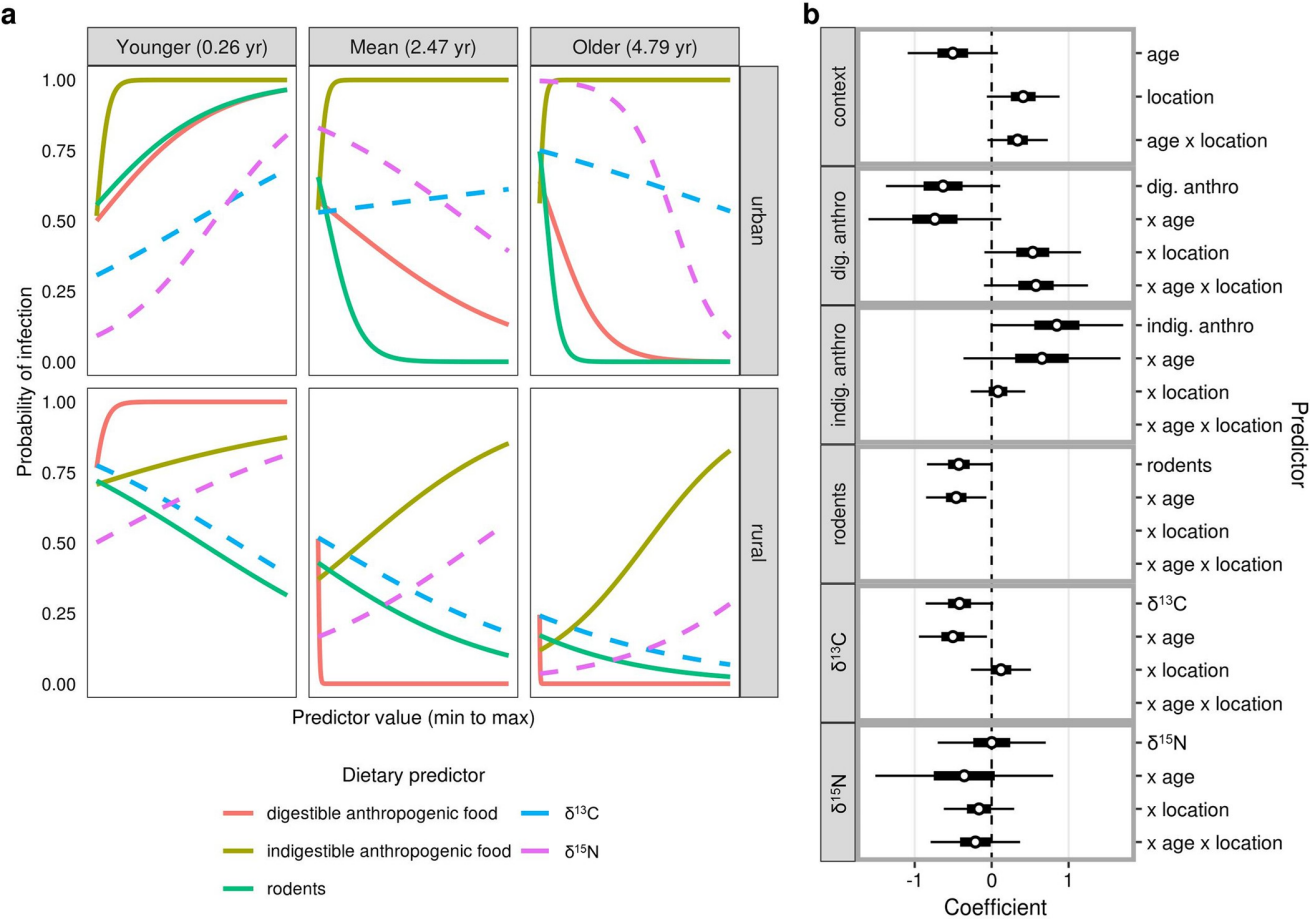
Dietary drivers of *E*. *multilocularis* infection status. (**a**) Three-way interactions between age, location, and each focal diet item (stomach contents [solid lines, measured in ml] and stable isotope values [dashed lines]). As in Fig 3, relationships are plotted separately for urban and rural coyotes at three different ages. All diet components were rescaled so that the x-axis extends from the minimum to maximum value for each item (digestible anthropogenic food, 0–360 ml; indigestible anthropogenic food, 0–160 ml; rodents, 0–325 ml; δ^13^C, -24.3–-19.2, δ^15^N, 5.2–10.8). Refer to **Table 1** for the results of likelihood ratio tests indicating the significance of these relationships. (**b**) Model-averaged coefficients for the top models predicting infection intensity. Each main effect is plotted followed by its two- and three-way interactions with other variables (indicated with an ‘x’). The coefficient for location reflects urban relative to rural coyotes. Coefficients were standardized by the partial standard deviation and weighted based on model weight prior to averaging. Thick and thin lines indicate 50% and 95% confidence intervals, respectively.

**Fig 5:**
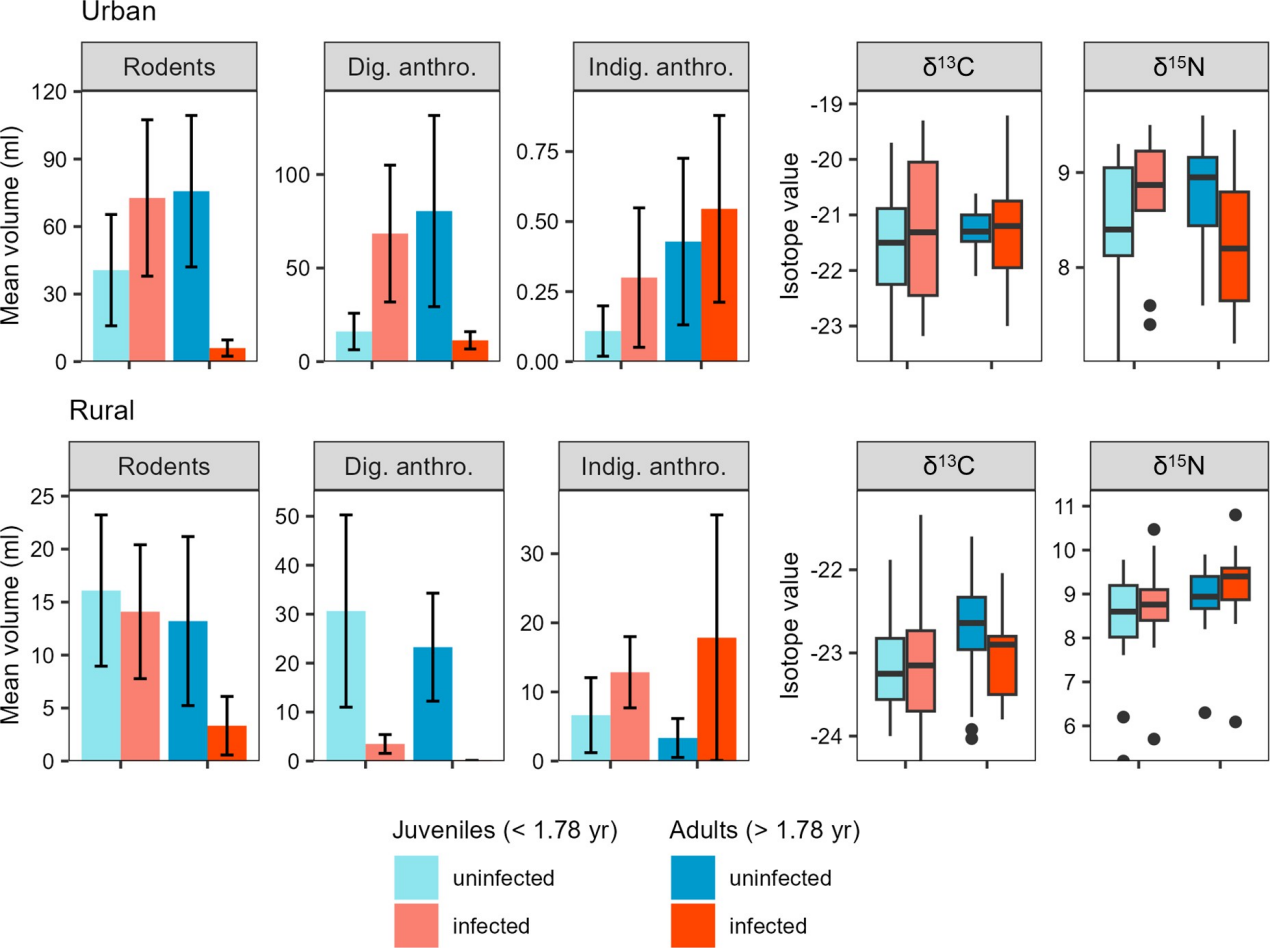
Coyote diet and stable isotope measures in relation to coyote infection status, age, and location (urban or rural). Note the different scales on each panel. In the bar graphs, error bars indicate the standard error. In the boxplots, the box indicates the median and interquartile range, the line represents the 95^th^ percentile, and the dots represent outliers. Coyotes were classified as juvenile or adult based on whether they were younger or older, respectively, than the median sample age (1.78 yr).

Despite the lack of clear univariate associations between *E*. *multilocularis* infection and diet, several dietary variables became significant predictors of infection status when accounting for their two- and three-way interactions with location and/or age. With respect to our initial hypotheses that either rodent or anthropogenic food consumption might drive infections, we found that rodent consumption was positively associated with infection in young (< 1 yr) urban coyotes, but negatively associated with infection in older (> 1 yr) urban coyotes and rural coyotes of all ages (**Figs 4A and 5; Table 1**). Digestible anthropogenic food consumption was positively associated with infection in younger coyotes from both urban and rural areas, but negatively associated with infection in older individuals (**Figs 4A and 5; Table 1**). Carbon stable isotope values (δ^13^C) followed the same trend as rodents, whereby long-term anthropogenic food consumption was positively associated with infection in younger, urban coyotes but negatively associated with infection in older coyotes and rural coyotes (**Figs 4A and 5; Table 1**).

These predictors and their interactions were retained in the nine top-ranked models predicting infection status from a suite of dietary variables (**Fig 4B; S3 Table** in **S3 Appendix**). Although neither indigestible anthropogenic food consumption nor δ^15^N values had significantly predicted infection in two- or three-way interaction models, these predictors and some of their associated interactions were retained in the top model set (**Fig 4B**). In these models, indigestible anthropogenic food consumption was associated with infection at all ages and in both locations, and long-term protein consumption (δ^15^N) was associated with infection in rural coyotes and young, urban coyotes but not older, urban coyotes (**Fig 4A**). Based on model-averaged coefficient values, digestible and indigestible anthropogenic food consumption had a stronger relationship with infection status than rodent consumption or stable isotope values (**Fig 4B**). The accuracy of the top-ranked models ranged from 65.1–73.1% in cross-validation tests, with Cohen’s kappa of 0.35–0.46 and McFadden’s pseudo-R^2^ of 0.34–0.39, suggesting that the models had a fair to moderate level of accuracy when predicting infection status from dietary variables and their interactions with age and location (**S3 Table** in **S3 Appendix**).

### Diet and infection intensity

**Fig 6:**
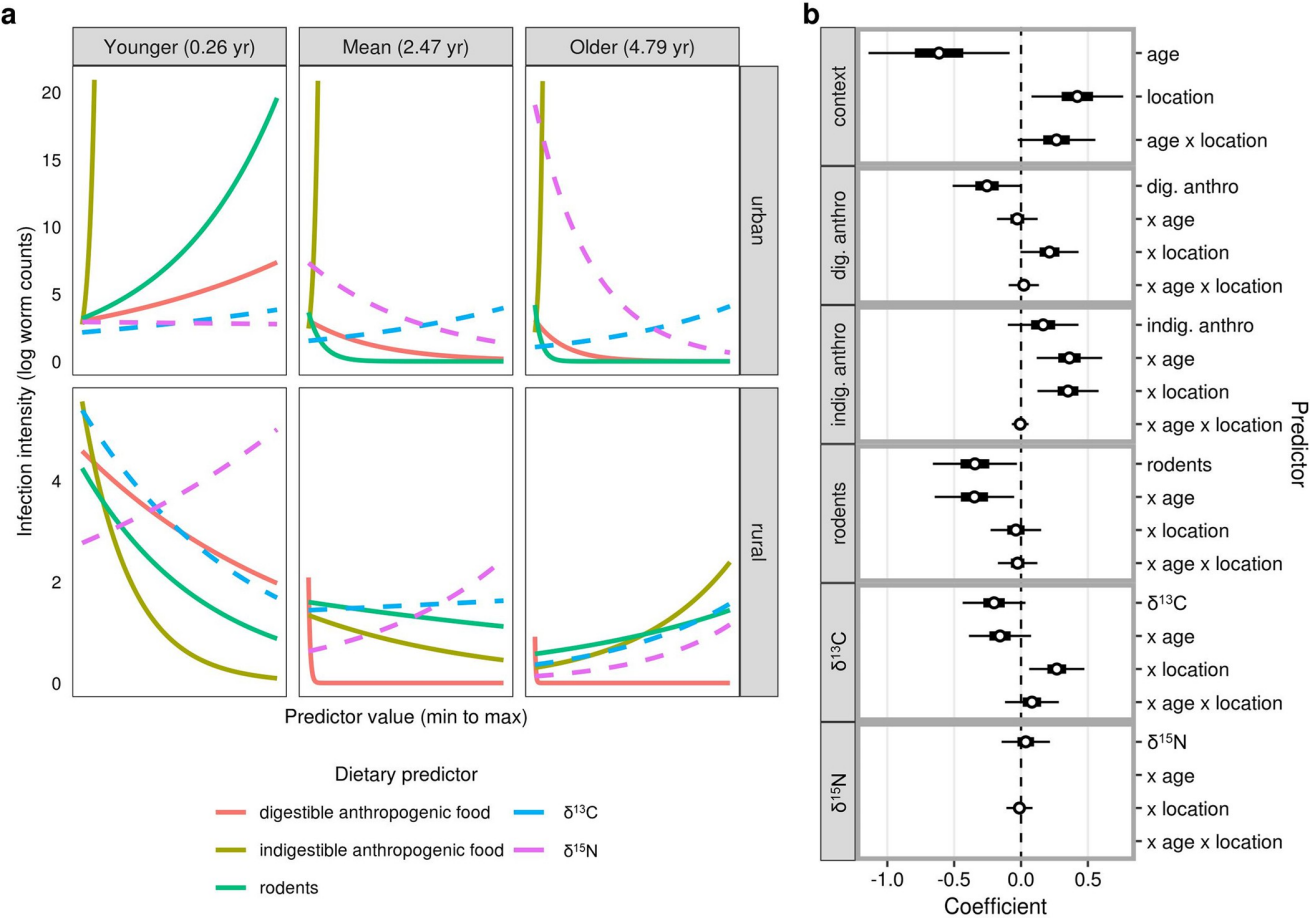
Dietary drivers of *E*. *multilocularis* infection intensity. (**a**) Three-way interactions between age, location, and each focal diet item (stomach contents [solid lines, measured in ml] and stable isotope values [dashed lines]). As in Figs 3 and 4, relationships are plotted separately for urban and rural coyotes at three different ages. All diet components were rescaled so that the x-axis extends from the minimum to maximum value for each item (see Fig 4). Refer to **Table 1** for the significance of these relationships. (**b**) Model-averaged coefficients for the top models predicting infection intensity. Each main effect is plotted followed by its two- and three-way interactions with other variables (indicated with an ‘x’). The coefficient for location reflects urban relative to rural coyotes. Coefficients were standardized by the partial standard deviation and weighted based on model weight prior to averaging. Thick and thin lines indicate 50% and 95% confidence intervals, respectively.

Dietary drivers of infection intensity largely mirrored the dietary drivers of infection status: digestible anthropogenic food and rodent consumption both predicted infection intensity in significant three-way interactions with location and age. As with models for infection status, both measures were strong predictors of infection intensity in younger, urban coyotes but had reduced or opposite effects in older coyotes and rural coyotes (**Fig 6A**). Indigestible anthropogenic food consumption was also a significant predictor of infection intensity as part of a three-way interaction (**Fig 6A; Table 1**); it most strongly predicted infection intensity in urban coyotes, though this was largely because one of the three highly infected urban coyotes (>10,000 worms) had consumed a small amount (3.5 ml) of indigestible anthropogenic food and most other urban coyotes had not consumed any (**S4 Table** in **S1 Appendix; S2 Fig** in **S3 Appendix**). δ^13^C exhibited a significant interaction with age (**Table 1**), whereby higher values were negatively associated with worm counts at young ages but positively associated with worm counts at older ages. All five dietary predictors and most two- and three-way interactions were preserved in the thirteen top-ranked models predicting infection intensity (**Fig 6B; S4 Table** in **S3 Appendix**); however, these models had root mean-squared errors from 2.86–4.03 (measured on a log-transformed scale of worm counts) and R^2^ values less than 0.14 (**S4 Table** in **S3 Appendix**), suggesting that models predicting infection intensity were generally less accurate than models predicting infection status.

## Discussion

Mitigating the spread and public health risk of a trophically transmitted parasite like *E*. *multilocularis* requires understanding the dietary drivers of *E*. *multilocularis* infection. We hypothesized that coyote diets would relate to increased *E*. *multilocularis* infection via two potential mechanisms: greater exposure to the parasite, indicated by greater consumption of rodent intermediate hosts, or greater susceptibility to infection, indicated by greater consumption of poor-quality anthropogenic food. Overall, we found limited evidence that these mechanisms act consistently across our entire sample pool: neither short- nor long-term measures of diet could universally discriminate between infected and uninfected coyotes, and the best overall predictor of infection status and intensity was a younger age. Nevertheless, when accounting for the effects of age, short-term measures of rodent and anthropogenic food consumption were the best dietary predictors of infection, though not always in the ways we expected. We found support for our initial hypotheses in young, urban coyotes: digestible and indigestible anthropogenic food consumption, rodent consumption, and δ^13^C and δ^15^N values all predicted *E*. *multilocularis* infection in this population. However, the effects of these predictors were more variable in rural coyotes and older coyotes and for models predicting infection intensity, suggesting that the relationship between diet and infection may be a complex function of habitat use, age and/or body condition, and other variables that we could not measure.

Our estimate of the overall prevalence of active *E*. *multilocularis* infections in Edmonton’s urban coyote population (56.1%) generally matches the previously published estimates of infection prevalence based on subsamples of our study population (65.2% by Luong et al. [26], 53% by Sugden et al. [27]), though we observed a higher prevalence among rural coyotes than in our previous study (43% here vs. 35% by Sugden et al. [27]). Some of these discrepancies could be associated with the different methods used to test for infections: in this study, we used a sensitive molecular test (qPCR) on samples taken from the jejunum, where *E*. *multilocularis* is most expected to occur [55,56]. Nevertheless, the prevalence of *E*. *multilocularis* in both the urban and rural populations studied here is notably higher than in other Canadian coyote populations and much higher than other Canadian urban areas for which data is available (Calgary, 21–29%; Winnipeg, 7%; see (**S6 Table** in **S1 Appendix** for data and references). Moreover, our qPCR results indicated that over 80% of urban coyotes carried *E*. *multilocularis* DNA (compared to 64% of rural coyotes). Although the qPCR-positive intestines with no visible worms presumably reflect an infection that is not producing viable eggs, the high DNA detection rate underscores the frequency with which urban coyotes in Edmonton may encounter, acquire, and potentially transmit *E*. *multilocularis* infections.

### Age-dependent immunity

With respect to contextual drivers of infection, we found that young age was the best overall predictor of *E*. *multilocularis* infections. This result agrees with previous reports of more *E*. *multilocularis* infections among young coyotes [33,67] and foxes [31,32], though not all studies have found an equivalent relationship between age and *E*. *multilocularis* infection [25,26]. Increased parasitism in juveniles is common across a range of host and parasite taxa because juveniles generally have naïve immune systems and expend more energy on growth than immunity [29,68]. However, by adulthood, many animals produce a measurable immune response to gastrointestinal parasites; for example, older sheep infected with gastrointestinal helminths produce more lymphocytes than infected younger sheep [69], and adult dogs infected with *E*. *multilocularis* have increased levels of serum immunoglobulin G against adult worms [70]. It is unlikely that canids become entirely immune to *E*. *multilocularis* infection [71], but a study of domestic dogs experimentally reinfected with *E*. *multilocularis* five times over one year showed a tremendous reduction in infection intensity, with ~90% fewer adult worms than never-exposed individuals [72,73].

Our results provide several lines of indirect evidence for increasing immunity with age. First, not only were young coyotes more likely to be infected, but coyotes that were qPCR-positive for *E*. *multilocularis* DNA without visible worms were, on average, 13.5 months older than coyotes that tested positive via both approaches, but 4.5 months younger than coyotes with no evidence of infection (**S2 Fig** in **S2 Appendix**). Second, infection intensities peaked at young ages: 73% of the coyotes with >1,000 scolexes in their intestines were less than 1 year old, and the three individuals with >10,000 scolexes were all less than 6 months old. Finally, the relationship that we observed between immune system investment (measured as spleen mass) and infection status at different ages may reflect age-dependent resistance or immunity to *E*. *multilocularis*. In young coyotes, larger spleens were associated with a lower probability of infection; for these individuals, a larger spleen may indicate greater investment in immune development [52] and therefore a greater ability to resist infection. Conversely, larger spleens were associated with a higher probability of infection in older animals, which could indicate the effect of a current infection: spleen size swells as the animal mounts an immune response, an effect that has been observed in several other species including geese [74] and various rodents [75].

The evidence of increased immunity with age in our coyote sample was not evident in urban coyotes, suggesting that urban coyotes were less able to develop or mount an effective age-dependent immune response. Whereas the likelihood and intensity of infection declined significantly with age in rural coyotes, urban coyotes were equally likely to be infected at all ages. We suspect that this absent signal of increasing immunity with age stems from the overall greater immune stress experienced by urban animals [5]. For example, genes for inflammatory pathways, antioxidant production, and toxicant neutralization have all been shown to be upregulated in urban wildlife compared to rural conspecifics [76,77], and these stresses may make an animal less adept at responding to an infection or parasite [78,79]. In addition, urban coyotes consumed more anthropogenic food than rural coyotes, which can further reduce immune function by leading to malnutrition or directly suppressing immune activity [80,81].

### Dietary drivers of infection

With respect to our diet-based hypotheses, we initially predicted that the higher *E*. *multilocularis* prevalence in urban coyotes could be a result of either increased exposure to the parasite via consumption of infected hosts or increased susceptibility to the parasite due to the negative health effects of consuming anthropogenic food. After accounting for the interactions with age driven by potential age-dependent immunity, we found support for both these hypotheses in young (< 1 yr) urban coyotes: both short- and long-term measures of rodent and anthropogenic food consumption predicted infection status in this population, and short-term measures of these items also predicted infection intensity. We suspect that urban coyotes that forage at compost sites (as described by Murray et al. [49]), which provide ideal habitat for voles and other rodents, consume high quantities of both rodents and anthropogenic food, thus increasing both their exposure and potential susceptibility to infection. Contrary to our expectations, our two hypothesized mechanisms could not be clearly disentangled and may act synergistically. Actually, similar concerns about the overlap between rodent habitat and anthropogenic food sources in relation to *E*. *multilocularis* have been voiced in Europe owing to the fact that both rodents and anthropogenic food attract foxes [82].

Intriguingly, neither rodent nor digestible anthropogenic food consumption continued to predict infection status or intensity in urban coyotes as they aged, even though the likelihood of infection did not decline with age. This finding opposes an age-independent positive correlation between rodent consumption and infection intensity in foxes [42]. Given that the maximum lifespan of adult *E*. *multilocularis* worms is approximately 7 months [83], older coyotes would still need to be consuming infected rodents to maintain active, detectable infections. Liccioli et al. [84] estimated that over 57% of urban coyotes in Calgary become reinfected following an initial infection, and Liccioli et al. [44] estimated that coyotes consume, on average, 1.05 infected intermediate hosts during the winter (our sample collection period). Our results suggest that older urban coyotes still consume enough infected intermediate hosts to maintain ongoing infections, even if their overall diet shifts to larger prey with age and increased rodent consumption no longer directly predicts infection. However, longitudinal studies using non-invasive or non-destructive approaches to track infection and diet over time would be needed to resolve this complex interplay between rodent consumption and age-related *E*. *multilocularis* exposure and infection.

We found more mixed evidence for our diet-based hypotheses in rural coyotes: short-term anthropogenic food consumption from stomach contents predicted infection only at the youngest (<0.5 yr) ages, and short-term rodent consumption did not predict infection at any age. Instead, indigestible anthropogenic food consumption predicted infection at all ages, though these relationships were not all statistically significant. The rural coyotes with the greatest amounts of indigestible anthropogenic food in their stomachs were not only more likely to be infected at higher worm burdens, but they were also younger coyotes captured closest to the Leduc regional landfill (**S3 Fig** in **S3 Appendix**). In this case, the relationship between digestible and indigestible anthropogenic food consumption and *E*. *multilocularis* infection in young coyotes may parallel the relationship observed in urban coyotes: young individuals that congregate and/or forage in areas where rodent habitat and anthropogenic food sources overlap (e.g., compost piles or garbage dumps) are more likely to be infected. It is not entirely clear why infected, young, rural coyotes would not also have consumed more rodents than uninfected coyotes, but it is possible that the overall reliance of juvenile coyotes on small prey items obscured this effect. In addition, plastic consumption has been associated with poorer nutrition, intestinal inflammation, and exposure to immune-compromising toxins [85], all of which may drive increased susceptibility to *E*. *multilocularis* infection without increased rodent consumption.

### Limitations

We acknowledge that our study is limited in its ability to determine how specific dietary components contribute to parasite presence or intensity in a population of wild carnivores. First, our method of sample collection may bias our data towards a younger overall age distribution because younger animals are more prone to being trapped, hit by cars, and coming into conflict with humans. The abundance of young coyotes in our study may therefore have inflated our population-wide measures of parasite prevalence and intensity [86,87], though we note that a previous sample of road-killed coyotes in Edmonton was not biased toward younger animals [88]. Second, we note that the likelihood of infection depends not only on the number of intermediate hosts consumed but also the prevalence of *E*. *multilocularis* among intermediate hosts. Given the paucity of available data on this topic—the most recent estimate of *E*. *multilocularis* prevalence in rodents comes from 1970, when Holmes et al. [89] observed a prevalence of 25% among rodents near Edmonton—our study design assumes a consistent prevalence across all habitats we sampled. Nevertheless, given the generally high prevalence of *E*. *multilocularis* among urban carnivores, we suggest that future studies should focus in parallel on the prevalence of *E*. *multilocularis* among intermediate hosts.

Finally, because *E*. *multilocularis* requires up to 60 days to mature in coyote intestines [44], our measures of diet do not directly align with timescale of infection. Stomach contents only reflect the last meal before death and could overrepresent food items that are less digestible [90]; conversely, stable isotopes are too coarse to reflect specific diet components, such as the distinction between rodents and other prey. This may partly explain why our models for infection intensity had limited predictive power. However, previous studies in Edmonton and elsewhere suggest that individual coyotes and other canids specialize over time and have reasonably consistent diets [35,60], in which case stomach contents may serve as an appropriate proxy for diet at the time of infection. Moreover, our two methods of diet analysis are some of the few methods available for assessing diet in wild carnivores, making them representative of the approaches that would also be taken by wildlife managers.

## Management implications

Although previous studies have considered coyote culling [91], rodent poisons [92], and anthelminthic coyote baits [93] as strategies for controlling parasite spread, these approaches are often laborious, expensive, and have achieved only mixed success at controlling *E*. *multilocularis* infections in foxes in Europe [94–99]. Moreover, culling programs can reduce the age distribution of coyote populations and increase litter sizes [100], which might worsen the prevalence of this parasite. Our results instead suggest that effective management of urban coyotes and *E*. *multilocularis* should focus on identifying the resources and habitats preferred by young coyotes, because this would correspond to where eggs are more likely to be shed in the environment. In addition, managerial approaches designed to prevent coyotes from accessing communal anthropogenic food sources (e.g., compost piles or garbage facilities) could reduce the spatial overlap between coyotes and rodents, which is the most likely reason for why both anthropogenic food and rodent consumption were associated with infection in young urban coyotes. Because the extended asymptomatic period of alveolar echinococcosis creates challenges in confirming sources of human exposure to *E*. *multilocularis* [24,101], mitigation strategies should also include public education that alerts citizens to the presence of *E*. *multilocularis*, the need to wash items that potentially come in contact with viable eggs (e.g., hands, toys, tools, garden produce), and the value of deworming dogs, especially those that consume rodents [102]. Overall, evidence that the more virulent European strain of *E*. *multilocularis* is now widespread in Canada and has infected over 20 people in Alberta [24] speaks to the need for more research on how best to limit infections in wildlife and minimize the risk of transmission to humans.

## Supporting information

S1 Appendix Supplementary methods and sample overview. Additional information on the statistical methods used for data analysis and the coyote sample used in this study. This appendix includes coyote demographic data (age, sex, year of collection, etc.), diet information (percent occurrence of difference diet items), and the full results of statistical tests related to these variables.
(DOCX)

S2 Appendix Analyses reproduced for qPCR-positive coyotes. For the analyses presented in the main manuscript text, we focused on coyotes that both (i) tested positive for *E*. *multilocularis* via qPCR and (ii) had visible evidence of *Echinococcus* scolexes in their intestines. To demonstrate that this decision did not substantively alter our conclusions, this appendix reproduces our analyses, except in these cases we considered all qPCR-positive coyotes as positive for *E*. *multilocularis*, regardless of whether scolexes were observed in the intestine.
(DOCX)

S3 Appendix Predictors of *E*. *multilocularis* infection. Full numerical results for (i) statistical tests for differences in infection status or intensity as a function of contextual variables or diet and (ii) generalized linear models predicting infection status or intensity as a function of these same variables. These data support Figs 3, 4 and 6 in the main manuscript text.
(DOCX)
