# Supplementary material for: Age-dependent relationships among diet, body condition, and Echinococcus multilocularis infection in urban coyotes
Source: PLoS One. 2023 Aug 30;18(8):e0290755. doi: 10.1371/journal.pone.0290755 (PMC10468061; doi:10.1371/journal.pone.0290755)
Supplement: S1 Appendix — Additional information on the statistical methods used for data analysis and the coyote sample used in this study. This appendix includes coyote demographic data (age, sex, year of collection, etc.), diet information (percent occurrence of difference diet items), and the full results of statistical tests related to these variables. (DOCX) [file pone.0290755.s001.docx]

**Age-dependent relationships among diet, body condition, and *Echinococcus multilocularis* infection in urban coyotes**

S. Sugden, D.K. Steckler, D. Sanderson, B. Abercrombie, D. Abercombie, M.A. Seguin, K. Ford, C.C. St. Clair

**S1 APPENDIX: Supplementary methods and sample overview**

Additional information on the statistical methods used for data analysis and the coyote sample used in this study. This appendix includes coyote demographic data (age, sex, year of collection, etc.), diet information (percent occurrence of difference diet items), and the full results of statistical tests related to these variables.

# SUPPLEMENTARY METHODS

### Data processing

We interpolated nine age measurements for which cementum images were inclusive. Ages were interpolated using a linear regression model with the remaining physiological measurements (i.e., mass, length, girth, and KFI) as predictors. Interpolated values were compared against the low-quality cementum images to ensure their relative accuracy.

### Quantitative real-time PCR (qPCR) analysis

We provided IDEXX Laboratories (West Sacramento, CA) with ~0.5 g scrapings taken from the jejunal mucosa of each coyote. Samples were stored at -20°C between collection and analysis. Quantitative real-time PCR (qPCR) was performed on a Roche LightCycler 480 system at IDEXX Laboratories using their commercially available *Echinococcus* RealPCR™ Panel, which uses proprietary forward and reverse primers and hydrolysis probes targeting a ribosomal RNA sequence between the *cox1* and *cox2* genes. Following the guidelines of the commercially available test, samples were considered qPCR-positive for *E. multilocularis* if the fluorescence of the sample crossed the threshold line before 40 cycles of PCR (i.e., CP < 40).

The qPCR reactions were performed with seven quality controls to ensure that results were not affected by the DNA extraction or PCR processes. These controls were: (1) PCR positive control; (2) PCR negative control; (3) DNA extraction negative control; (4) DNA pre-analytic quality control targeting the host 18S rRNA gene complex; (5) RNA pre-analytic quality control targeting the host 18S rRNA gene complex; (6) an internal positive control spiked into the lysis solution to monitor the extraction efficiency; and (7) a control for monitoring environmental contamination.

Nine samples could not be used for qPCR analysis because their intestines had already been destructively sampled for previous research projects. Worm counts for these samples were measured and reported by Luong et al. (1) using an equivalent method to the approach described here, and Sugden et al. (2) performed conventional PCR tests for *E. multilocularis* in this samples. We used these two sources of data to define “biologically active” infections as we did for the remainder of the coyote samples (i.e., positive PCR test with non-zero worm counts). However, these nine samples were excluded from the qPCR-specific analyses provided in **Appendix S2**.

### Statistical methods

*Sample weighting*. Given the uneven sample sizes of urban and rural coyotes in our study, we chose to weight urban samples for all regression analysis so that urban and rural populations appeared equal in the model. This additional weight was particularly important for ensuring that interaction-based models adequately modeled interactions with coyote location. Thus, all urban coyotes were assigned a weight of $\frac{71}{41}=1.73$. We compared results from weighted models to results from unweighted models to ensure that this approach did not substantially alter the magnitude or direction of our conclusions (see **Appendix S3**).

*Univariate models*. We validated the results of all univariate regression models using chi-squared tests, Wilcox rank-sum tests, and Spearman’s rank correlations. Specifically, relationships between categorical variables (location, sex) and infection status were tested with chi-squared tests; relationships between continuous variables (age, condition, stomach contents, stable isotopes) and infection status were tested with Wilcox rank-sum tests; relationships between categorical variables and infection intensity were tested with Wilcox rank-sum tests; and relationships between continuous variables and infection intensity were tested with Spearman’s correlation. We chose non-parametric tests (Wilcox rank-sum, Spearman’s R) over their parametric alternatives (Student’s t-test, Pearson’s R) due to the uneven sample sizes and heteroskedastic distributions most predictors, as assessed using Levene’s test. We used a significance threshold of p < 0.05 for all comparisons.

*Interaction models*. We also tested interaction effects to determine whether the effect of a focal predictor (stomach contents, stable isotopes, body condition) differed between urban and rural areas and/or across different ages. For each response/predictor pair, we ran three additional models, allowing our focal variable $fv$ (for example, the volume of rodents in the stomach) to interact with (*i*) age, (*ii*) location, and (*iii*) the two-way interaction between age and location, as shown below:

$$infection=age*fv$$

$$infection=location*fv$$

$$infection=age*location*fv$$

These models were run for both infection status and intensity. Because age and location were some of the strongest predictors of infection, we determined the significance of each interaction using a likelihood ratio test in which each model was compared to the model in which the focal variable $fv$ did not appear. In this way, we tested whether the focal variable (some dietary measure) significantly improved upon the predictive power of age, location, or the two-way interaction between age and location.

*Multi-diet-component models*. Finally, we allowed multiple measures of diet to appear in the same model so that we could determine the overall best dietary predictors of infection status and intensity. For these models, we chose only the dietary predictors that explicitly tested our hypotheses about rodent and/or anthropogenic food consumption driving infections. Thus, our final models included three stomach content measurements (rodents, digestible anthropogenic food, and indigestible anthropogenic food) and both stable isotope measurements (d13C and d15N). Given the number of significant interactions with age and/or location, we chose to evaluate all model subsets generated from the three-way interactions between age, location, and each dietary component. Our global model, run once for infection status and once for infection intensity, was:

$$infection=age*location*(dig. anthro+indig. anthro+rodents+d13C+d15N)$$

*Model evaluation*. We quantified the predictive accuracy of the top-ranked models using Cohen’s kappa (for the logistic regression) and root mean-squared error (for the negative binomial regression) calculated from k-fold cross-validation with k = 5.

*Analysis of qPCR-positive infections.* We repeated all statistical analyses using qPCR-positive infections instead of “biologically active” infections. Those results are presented in **Supplementary Note #S5**. However, we chose to focus on the results for biologically active infections because we believe these cases are more important from an ecological and management standpoint. For example, management strategies targeted towards animals with active infections—in other words, animals that are more likely to be shedding eggs into the environment—will be more likely to mitigate the public health risks of *E. multilocularis*.

# REFERENCES

1. Luong LT, Chambers JL, Moizis A, Stock TM, St Clair CC. Helminth parasites and zoonotic risk associated with urban coyotes (*Canis latrans*) in Alberta, Canada. J Helminthol. 2018:1-5.

2. Sugden S, Sanderson D, Ford K, Stein LY, St Clair CC. An altered microbiome in urban coyotes mediates relationships between anthropogenic diet and poor health. Sci Rep. 2020;10(1):22207.

# SAMPLE OVERVIEW

**
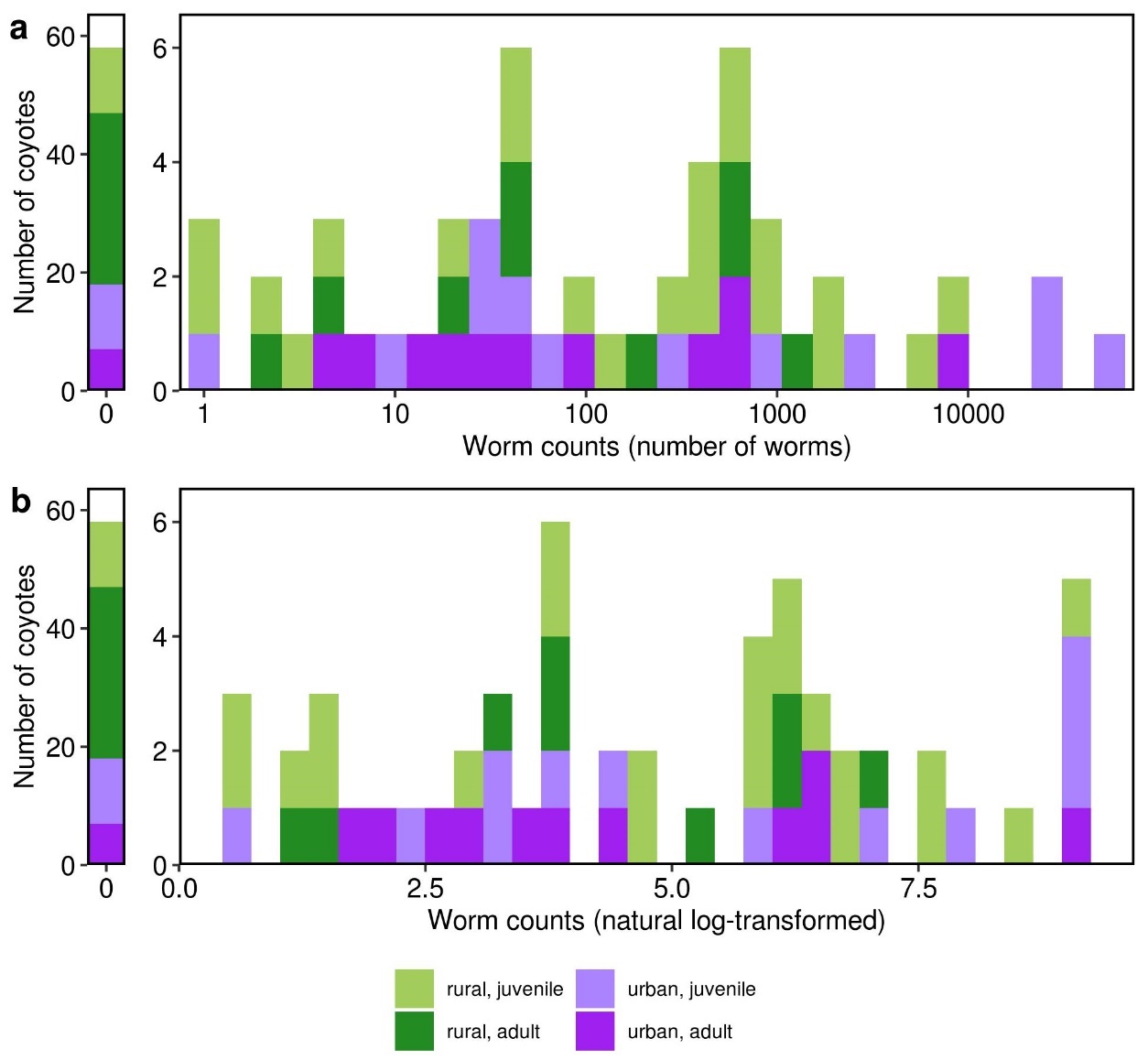
**

#### S1 Figure.

Histograms showing the distribution of *E. multilocularis* worm counts in coyote intestines (**a**) before and (**b**) after a natural log transformation. Coyotes are colored based on their location of capture and their age; we defined juveniles as ≤ 1.78 yr based on the median age of our sample. Note that uninfected coyotes (i.e., coyotes with zero worms in their intestines) are presented on a separate y-axis, and the x-axis for absolute worm counts is shown on a log-10 scale for ease of visualization.


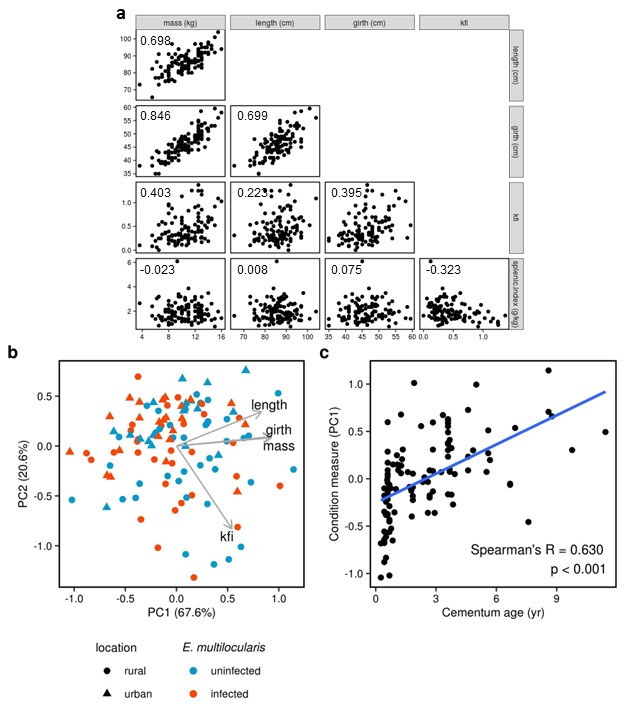


#### S2 Figure.

To generate a single metric for body condition, we performed principal components analysis using body mass, girth, length, and the kidney fat index (KFI). All variables were mean-centered and standardized prior to the ordination. (**a**) Spleen mass was excluded from this metric because it was not correlated with the other measures; numbers in each panel indicate Pearson’s correlation coefficient (**b**) The first principal component was significantly correlated with all four input measures; correlation coefficients are provided in **Table S#.** (**c**) The composite condition metric was significantly correlated with age; as expected, only healthy animals live to older ages.


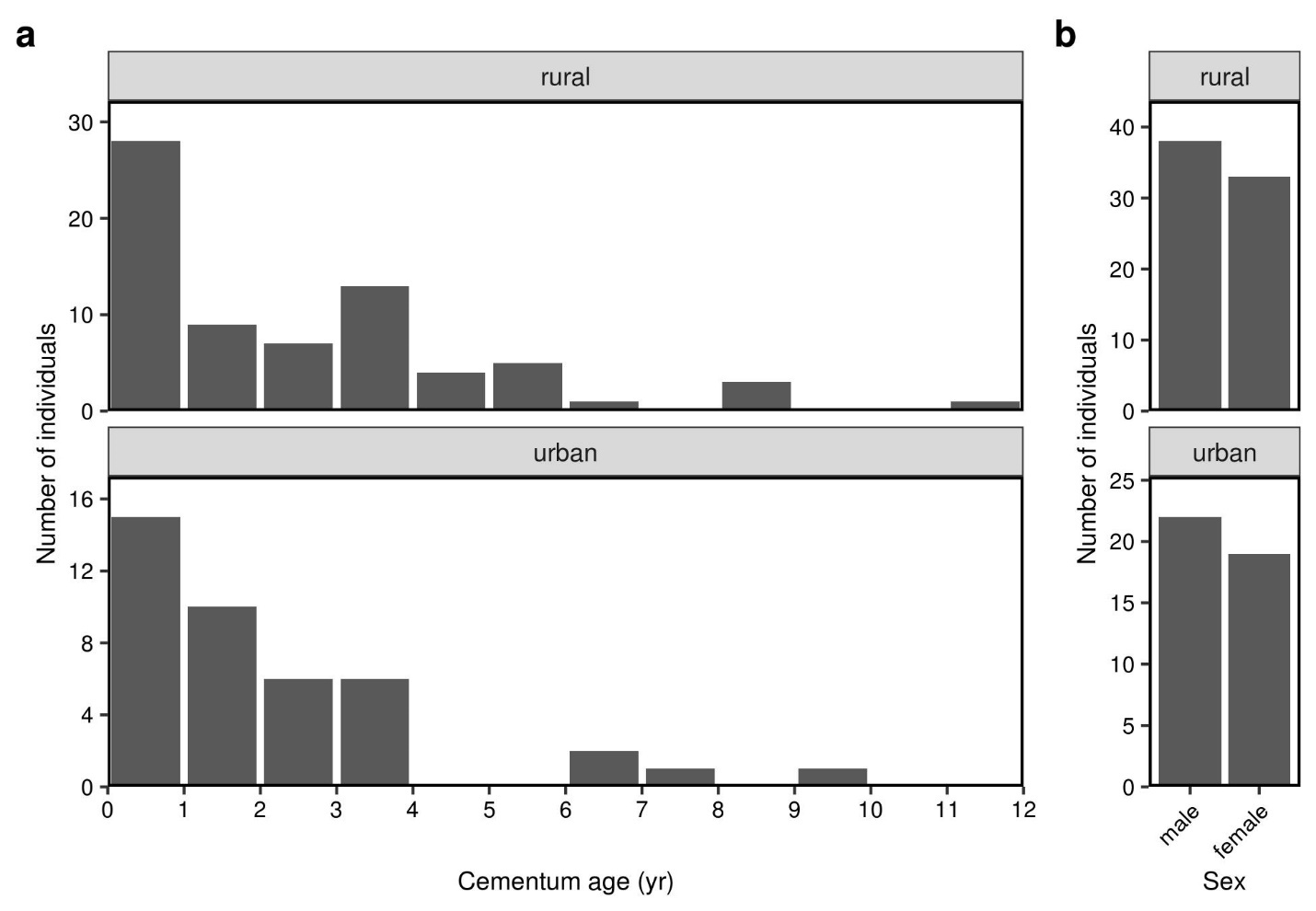


#### S3 Figure.

Coyote age and sex distribution. Note the different scales for rural and urban coyotes due to the different sample sizes.

#### S1 Table.

Coyote carcasses collected for this study, distinguished by location (urban/rural), collection year, sex, and mode of death (roadkill, lethally managed, or trapped as part of population management efforts).

|  | Year | Sex | |  | Mode of death | | |
| --- | --- | --- | --- | --- | --- | --- | --- |
|  |  | Male | Female |  | roadkill | trapped | conflict |
| **urban (n = 41)** | 2017 | 2 | 1 |  | 3 | 0 | 0 |
|  | 2018 | 5 | 7 |  | 8 | 2 | 2 |
|  | 2019 | 12 | 9 |  | 18 | 0 | 3 |
|  | 2020 | 3 | 2 |  | 4 | 1 | 0 |
|  | **Total** | **22** | **19** |  | **33** | **3** | **5** |
| **rural (n = 71)** | 2017 | 26 | 19 |  | 1 | 44 | 0 |
|  | 2018 | 9 | 11 |  | 2 | 17 | 1 |
|  | 2019 | 2 | 0 |  | 0 | 0 | 2 |
|  | 2020 | 1 | 3 |  | 0 | 1 | 3 |
|  | **Total** | **38** | **33** |  | **3** | **62** | **6** |

#### S2 Table.

Axis loadings for our composite metric of body condition. To generate a single metric of body condition, we performed principal components analysis using body mass, girth, length, and the kidney fat index (KFI). All variables were mean-centered and standardized prior to the ordination. The table shows the Pearson correlation between each of the initial variables and the two resulting PCA axes; the ordination itself is shown in **Fig. S2**.

|  | PC1 | |  | PC2 | |
| --- | --- | --- | --- | --- | --- |
| variable | R | p |  | R | p |
| mass | 0.928 | **< 0.001** |  | 0.083 | 0.383 |
| girth | 0.927 | **< 0.001** |  | 0.094 | 0.325 |
| length | 0.831 | **< 0.001** |  | 0.344 | **< 0.001** |
| kfi | 0.543 | **< 0.001** |  | -0.830 | **< 0.001** |

#### S3 Table.

Percent occurrence of the different food items measured in this study, for all coyotes as well as for urban, rural, juvenile, and adult coyotes separately. Occurrence was measured as the percentage of stomachs that contained the food item. Empty stomachs were excluded from these calculations.

|  |  |  |  | location | | | | |  | age class | | | | |
| --- | --- | --- | --- | --- | --- | --- | --- | --- | --- | --- | --- | --- | --- | --- |
|  | all samples (N=101) | |  | urban (N=37) | |  | rural (N=64) | |  | juvenile (N=53) | |  | adult (N=48) | |
| Stomach contents | N | freq (%) |  | N | freq (%) |  | N | freq (%) |  | N | freq (%) |  | N | freq (%) |
| anthropogenic food | 60 | 59.4 |  | 22 | 59.5 |  | 38 | 59.4 |  | 35 | 66.0 |  | 25 | 52.1 |
| digestible | 50 | 49.5 |  | 21 | 56.8 |  | 29 | 45.3 |  | 27 | 50.9 |  | 23 | 47.9 |
| indigestible | 37 | 36.6 |  | 11 | 29.7 |  | 26 | 40.6 |  | 23 | 43.4 |  | 14 | 29.2 |
|  |  |  |  |  |  |  |  |  |  |  |  |  |  |  |
| prey items | 88 | 87.1 |  | 29 | 78.4 |  | 59 | 92.2 |  | 46 | 86.8 |  | 42 | 87.5 |
| rodent | 50 | 49.5 |  | 23 | 62.2 |  | 27 | 42.2 |  | 29 | 54.7 |  | 21 | 43.8 |
| vole | 34 | 33.7 |  | 18 | 48.6 |  | 16 | 25.0 |  | 20 | 37.7 |  | 14 | 29.2 |
| unidentified | 13 | 12.9 |  | 4 | 10.8 |  | 9 | 14.1 |  | 9 | 17.0 |  | 4 | 8.3 |
| pocket gopher | 6 | 5.9 |  | 5 | 13.5 |  | 1 | 1.6 |  | 3 | 5.7 |  | 3 | 6.3 |
| deer mouse | 4 | 4.0 |  | 3 | 8.1 |  | 1 | 1.6 |  | 3 | 5.7 |  | 1 | 2.1 |
| shrew | 1 | 1.0 |  | 0 | 0.0 |  | 1 | 1.6 |  | 0 | 0.0 |  | 1 | 2.1 |
| muskrat | 1 | 1.0 |  | 0 | 0.0 |  | 1 | 1.6 |  | 0 | 0.0 |  | 1 | 2.1 |
| meso-mammal | 33 | 32.7 |  | 11 | 29.7 |  | 22 | 34.4 |  | 15 | 28.3 |  | 18 | 37.5 |
| ungulate | 36 | 35.6 |  | 2 | 5.4 |  | 34 | 53.1 |  | 15 | 28.3 |  | 21 | 43.8 |
| bird | 25 | 24.8 |  | 10 | 27.0 |  | 15 | 23.4 |  | 19 | 35.8 |  | 6 | 12.5 |
| insects | 7 | 6.9 |  | 5 | 13.5 |  | 2 | 3.1 |  | 6 | 11.3 |  | 1 | 2.1 |
|  |  |  |  |  |  |  |  |  |  |  |  |  |  |  |
| vegetation | 62 | 61.4 |  | 26 | 70.3 |  | 36 | 56.3 |  | 34 | 64.2 |  | 28 | 58.3 |
| herbaceous | 69 | 68.3 |  | 24 | 64.9 |  | 45 | 70.3 |  | 38 | 71.7 |  | 31 | 64.6 |
| woody | 32 | 31.7 |  | 11 | 29.7 |  | 21 | 32.8 |  | 20 | 37.7 |  | 12 | 25.0 |
|  |  |  |  |  |  |  |  |  |  |  |  |  |  |  |
| fruit | 11 | 10.9 |  | 5 | 13.5 |  | 6 | 9.4 |  | 10 | 18.9 |  | 1 | 2.1 |

#### S4 Table.

Significant differences in diet and contextual variables between locations. Because many dietary measures demonstrated heteroskedastic distributions, differences were assessed using Welch’s t-test.

|  |  | urban | rural | Welch's t-test (urban vs. rural) | |
| --- | --- | --- | --- | --- | --- |
| Variable |  | mean (sd) | mean (sd) | t | p |
| age |  | 2.2 (2.2) | 2.63 (2.39) | 0.913 | 0.342 |
| body condition |  | -0.1 (0.4) | 0.06 (0.45) | 3.824 | **0.054** |
| KFI |  | 0.29 (0.18) | 0.55 (0.32) | 30.313 | **< 0.001** |
| spleen size |  | 2.47 (0.82) | 1.7 (0.6) | 27.364 | **< 0.001** |
|  |  |  |  |  |  |
|  |  | Food volume (ml) | | Welch's t-test (urban vs. rural) | |
|  |  | urban | rural |  |  |
| Stomach contents |  | mean (sd) | mean (sd) | t | p |
| total food volume |  | 106.3 (127.43) | 229.1 (399.86) | 5.695 | **0.019** |
| diet diversity |  | 0.35 (0.34) | 0.40 (0.39) | 0.581 | 0.448 |
|  |  |  |  |  |  |
| anthropogenic food |  | 39.85 (88.04) | 23.92 (55.47) | 1.092 | 0.300 |
| digestible |  | 41.12 (91.55) | 15.34 (46.67) | 2.829 | **0.099** |
| indigestible |  | 0.34 (0.81) | 8.63 (25.83) | 7.313 | **0.009** |
| prey items |  | 62.85 (100.36) | 200.24 (402.67) | 7.463 | **0.008** |
| rodent |  | 46.72 (87.78) | 12.68 (33.22) | 5.697 | **0.021** |
| meso-mammal |  | 14.59 (53.26) | 28.35 (98.80) | 0.917 | 0.340 |
| ungulate |  | 0.44 (1.98) | 153.94 (397.36) | 10.595 | **0.002** |
| bird |  | 0.92 (3.33) | 5.27 (34.12) | 1.134 | 0.291 |
| insects |  | 0.18 (0.95) | 0 (0.02) | 1.409 | 0.242 |
| vegetation |  | 1.18 (2.57) | 1.46 (3.23) | 0.247 | 0.620 |
| herbaceous |  | 0.73 (1.72) | 2.72 (6.64) | 5.678 | **0.019** |
| woody |  | 0.45 (1.45) | 0.62 (2.14) | 0.272 | 0.603 |
| fruit |  | 0.54 (2.63) | 0.99 (7.16) | 0.233 | 0.631 |
|  |  |  |  |  |  |
| Stable isotopes |  |  |  |  |  |
| d13C |  | -21.36 (1.14) | -22.94 (0.66) | 65.925 | **< 0.001** |
| d15N |  | 8.55 (0.71) | 8.8 (1) | 2.455 | 0.120 |

#### S5 Table.

Significant differences in diet and contextual variables between age groups. Because many dietary measures demonstrated heteroskedastic distributions, differences were assessed using Welch’s t-test (for binary age categories) and Spearman’s correlation (for the continuous measure of age).

|  |  | juvenile | adult | Welch's t-test (juvenile vs. adult) | | Spearman's R (continuous age) | |
| --- | --- | --- | --- | --- | --- | --- | --- |
| Variable |  | mean (sd) | mean (sd) | t | p | R | p |
| age |  | 0.74 (0.35) | 4.21 (2.15) | 141.418 | **< 0.001** |  |  |
| body condition |  | -0.22 (0.38) | 0.22 (0.37) | 39.105 | **< 0.001** | 0.630 | **< 0.001** |
| KFI |  | 0.39 (0.28) | 0.52 (0.31) | 5.789 | **0.018** | 0.216 | **0.022** |
| spleen size |  | 1.91 (0.68) | 2.06 (0.86) | 1.134 | 0.289 | 0.086 | 0.366 |
|  |  |  |  |  |  |  |  |
|  |  | Food volume (ml) | | Welch's t-test (juvenile vs. adult) | | Spearman's R (continuous age) | |
|  |  | juvenile | adult |  |  |  |  |
| Stomach contents |  | mean (sd) | mean (sd) | t | p | R | p |
| total food volume |  | 145.6 (201.12) | 222.7 (423.03) | 1.517 | 0.222 | -0.072 | 0.453 |
| diet diversity |  | 0.45 (0.4) | 0.32 (0.33) | 3.261 | **0.074** | -0.206 | **0.030** |
|  |  |  |  |  |  |  |  |
| anthropogenic food |  | 31.66 (70.45) | 27.85 (68.57) | 0.084 | 0.772 | -0.180 | **0.057** |
| digestible |  | 25.21 (69.42) | 24.34 (66.05) | 0.005 | 0.946 | -0.114 | 0.231 |
| indigestible |  | 6.44 (17.74) | 4.76 (23.78) | 0.180 | 0.673 | -0.187 | **0.049** |
| prey items |  | 109.45 (200.04) | 190.44 (423.38) | 1.675 | 0.199 | -0.022 | 0.821 |
| rodent |  | 32.26 (71.5) | 18.02 (48.38) | 1.524 | 0.220 | -0.214 | **0.023** |
| meso-mammal |  | 26.37 (101.37) | 20.26 (65.43) | 0.144 | 0.705 | -0.038 | 0.690 |
| ungulate |  | 44.65 (171) | 150.84 (420.84) | 3.060 | 0.084 | 0.172 | **0.069** |
| bird |  | 6.03 (37.42) | 1.33 (9.35) | 0.834 | 0.365 | -0.327 | **< 0.001** |
| insects |  | 0.13 (0.81) | 0 (0.01) | 1.451 | 0.234 | -0.258 | **0.006** |
| vegetation |  | 1.25 (2.39) | 1.46 (3.51) | 0.147 | 0.702 | -0.088 | 0.354 |
| herbaceous |  | 1.31 (2.47) | 2.67 (7.29) | 1.747 | 0.191 | -0.065 | 0.499 |
| woody |  | 0.63 (2.00) | 0.48 (1.83) | 0.171 | 0.680 | -0.195 | **0.039** |
| fruit |  | 1.65 (8.31) | 0 (0.01) | 2.213 | 0.143 | -0.242 | **0.010** |
|  |  |  |  |  |  |  |  |
| Stable isotopes |  |  |  |  |  |  |  |
| d13C |  | -22.45 (1.27) | -22.27 (1.01) | 0.627 | 0.430 | 0.161 | **0.090** |
| d15N |  | 8.6 (0.96) | 8.82 (0.85) | 1.686 | 0.197 | 0.162 | **0.088** |

#### S6 Table.

*E. multilocularis* infection prevalence in different regions and cities in Canada, as reported by various studies. Prevalence estimates from Sugden et al. (2) and Luong et al. (1) were based on subsamples of the coyote population used in this study.

| **Reference** | **Year** | **Location** | **Location** | **# tested** | **# positive** | **Prevalence (%)** | **detection method** | **material** |
| --- | --- | --- | --- | --- | --- | --- | --- | --- |
| This study | 2023 | urban | Edmonton, AB | 41 | 33 | 80.5 | qPCR | intestine |
|  |  |  |  | 41 | 23 | 56.1 | morphology | intestine |
| Sugden et al. | 2020 | urban | Edmonton, AB | 30 | 16 | 53.3 | PCR | intestine / feces |
| Luong et al. | 2018 | urban | Edmonton, AB | 15 | 10 | 66.7 | morphology | intestine |
| Gesy et al. | 2014 | urban | Edmonton, AB | 5 | 5 | 100.0 | morphology / PCR | intestine |
|  |  |  | Saskatoon, SK | 6 | 6 | 100.0 | morphology / PCR | intestine |
| Catalano et al. | 2012 | urban | Edmonton, AB | 8 | 5 | 62.5 | morphology | intestine |
|  |  |  | Calgary, AB | 83 | 17 | 20.5 | morphology | intestine |
| Liccioli et al. | 2014 | urban | Calgary, AB | 385 | 82 | 21.3 | PCR | feces |
| Liccioli et al. | 2012 | urban | Calgary, AB | 61 | 18 | 29.5 | morphology / PCR | intestine |
| Tse et al. | 2019 | urban | Winnipeg, MB | 122 | 9 | 7.4 | PCR | feces |
|  |  |  |  |  |  |  |  |  |
| This study | 2023 | rural | Alberta | 71 | 45 | 63.4 | qPCR | intestine |
|  |  |  |  | 71 | 31 | 43.7 | morphology | intestine |
| Sugden et al. | 2020 | rural | Alberta | 65 | 23 | 35.4 | PCR | intestine / feces |
| Kolapo et al. | 2021 | rural | Saskatchewan | 208 | 150 | 72.1 | coproPCR | intestine |
|  | 2021 | rural | Quebec | 74 | 1 | 1.4 | coproPCR | intestine |
| Gesy et al. | 2014 | rural | Quesnel, BC | 27 | 10 | 37.0 | morphology / PCR | intestine |
|  |  | rural | Alberta | 6 | 4 | 66.7 | morphology / PCR | intestine |
|  |  | rural | Saskatchewan | 4 | 3 | 75.0 | morphology / PCR | intestine |
| Kotwa et al. | 2019 | rural | Ontario | 416 | 100 | 24.0 | qPCR | intestine |
| Schurer et al. | 2018 | rural | Quebec | 77 | 0 | 0.0 | PCR | intestine |
