# Supplementary material for: Age-dependent relationships among diet, body condition, and Echinococcus multilocularis infection in urban coyotes
Source: PLoS One. 2023 Aug 30;18(8):e0290755. doi: 10.1371/journal.pone.0290755 (PMC10468061; doi:10.1371/journal.pone.0290755)
Supplement: S2 Appendix — For the analyses presented in the main manuscript text, we focused on coyotes that both (i) tested positive for E. multilocularis via qPCR and (ii) had visible evidence of Echinococcus scolexes in their intestines. To demonstrate that this decision did not substantively alter our conclusions, this appendix reproduces our analyses, except in these cases we considered all qPCR-positive coyotes as positive for E. multilocularis, regardless of whether scolexes were observed in the intestine. (DOCX) [file pone.0290755.s002.docx]

**Age-dependent relationships among diet, body condition, and *Echinococcus multilocularis* infection in urban coyotes**

S. Sugden, D.K. Steckler, D. Sanderson, B. Abercrombie, D. Abercrombie, M.A. Seguin, K. Ford, C.C. St. Clair

**S2 APPENDIX: Analyses reproduced for qPCR-positive coyotes**

For the analyses presented in the main manuscript text, we focused on coyotes that both (i) tested positive for E. multilocularis via qPCR and (ii) had visible evidence of Echinococcus scolexes in their intestines. To demonstrate that this decision did not substantively alter our conclusions, this appendix reproduces our analyses, except in these cases we considered all qPCR-positive coyotes as positive for E. multilocularis, regardless of whether scolexes were observed in the intestine.


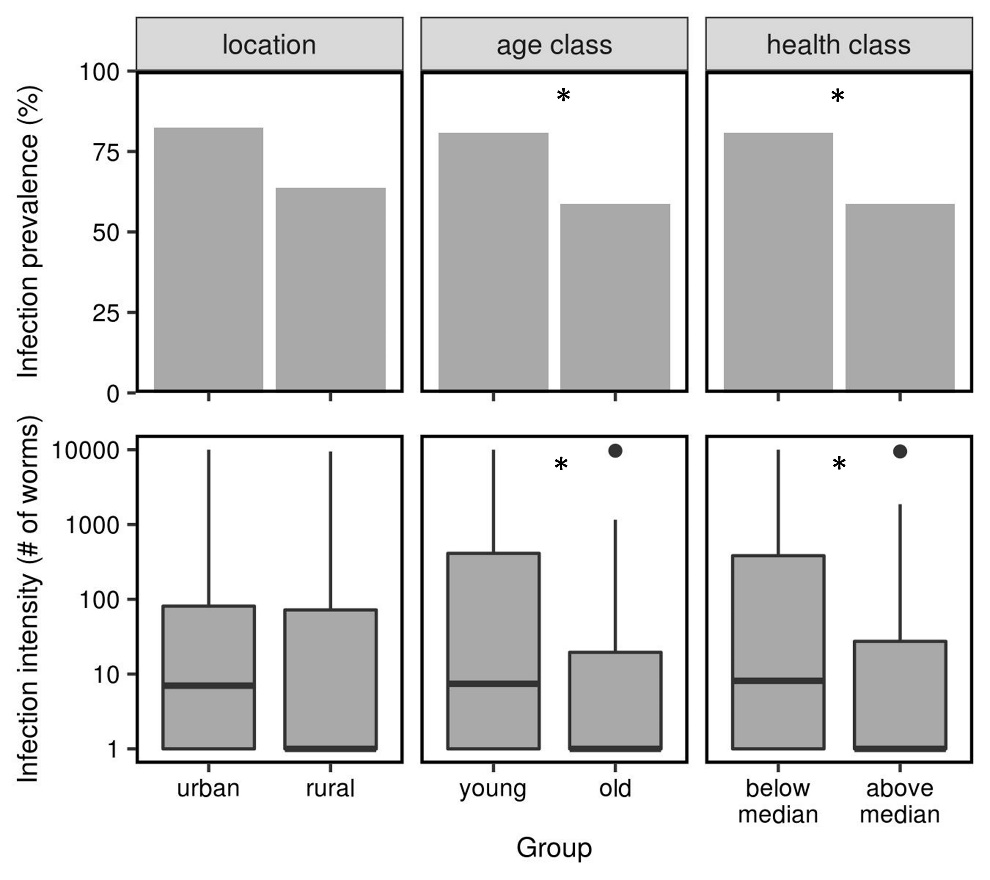


#### S1 Figure.

Figure 1 reproduced based on qPCR-positive infections instead of biologically active infections. Asterisks (*) indicate significant differences (p < 0.05) between groups.


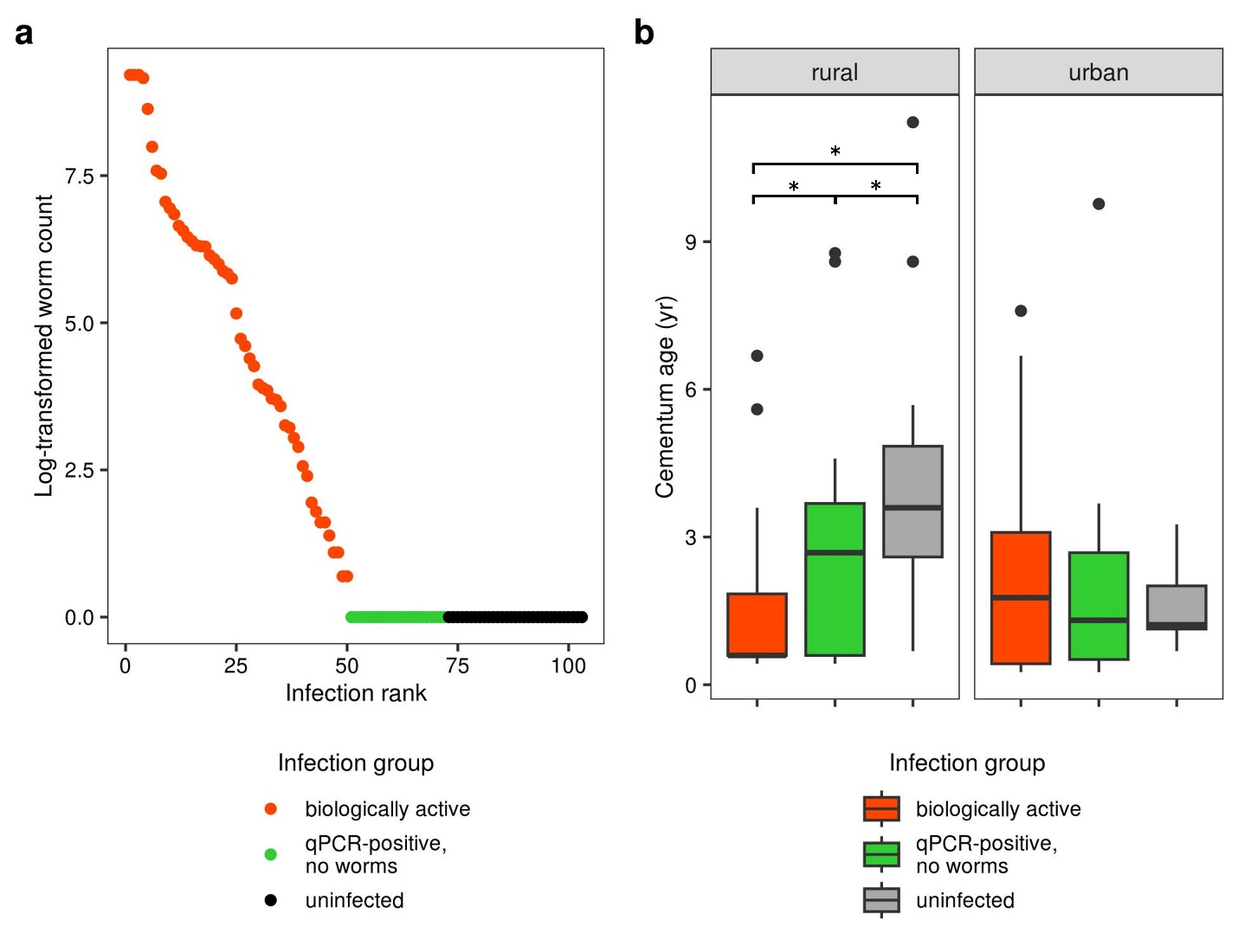


#### S2 Figure.

(**a**) Of the 101 samples that were tested via qPCR, 22 individuals tested positive via qPCR but had no evidence of worms in their intestine. (**b**) Rural coyotes that tested qPCR-positive but had no evidence of worms in their stomach were generally an intermediate age between those that tested positive via both approaches (qPCR-positive and non-zero worm count) and those that tested negative via both approaches (qPCR-negative and zero worms). This relationship was not observed in urban coyotes. Asterisks indicate significant (p < 0.05) differences assessed using Tukey’s *post hoc* test.


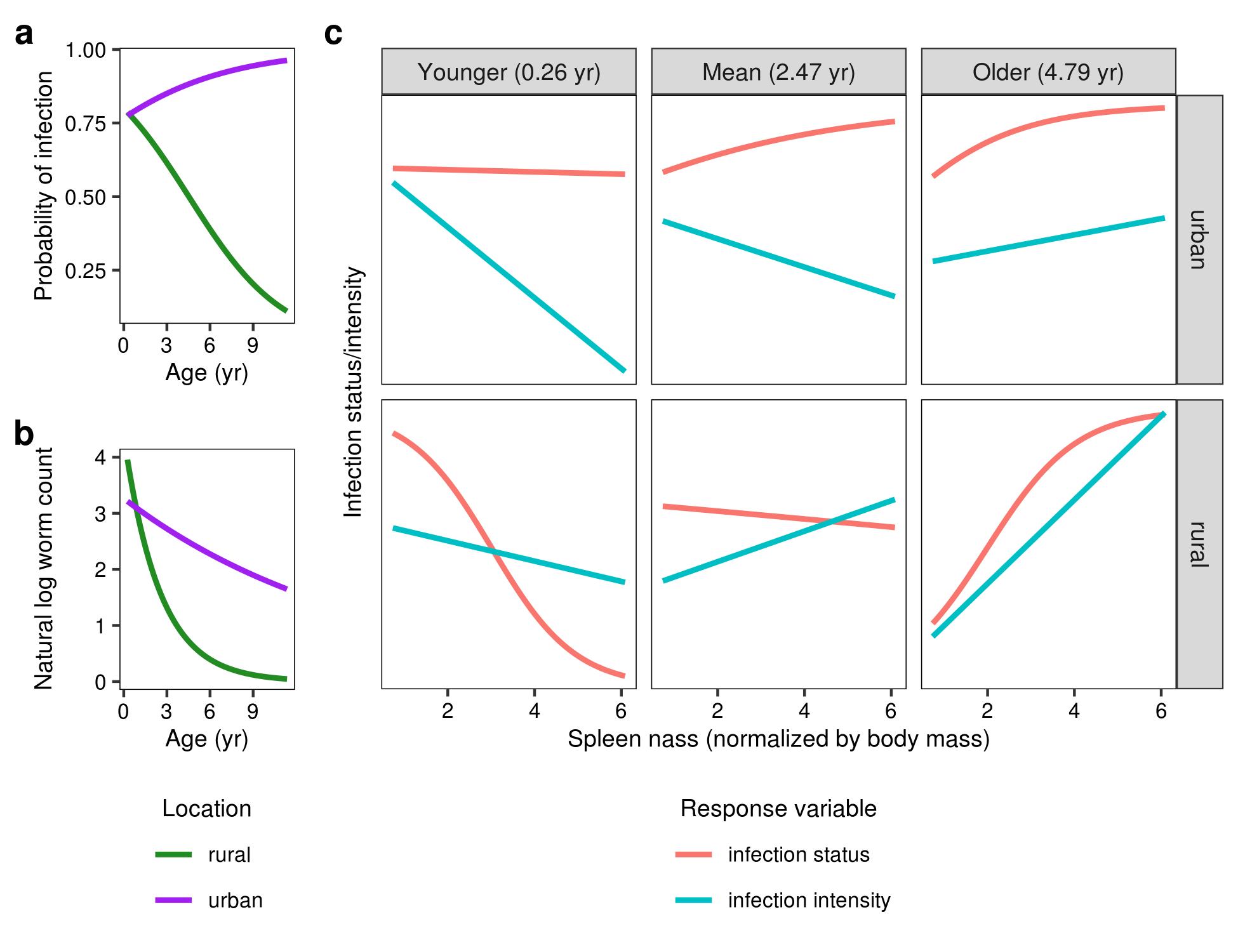


#### S3 Figure.

Figure 2 reproduced based on qPCR-positive infections instead of biologically active infections. See Fig. 2 for a complete legend.


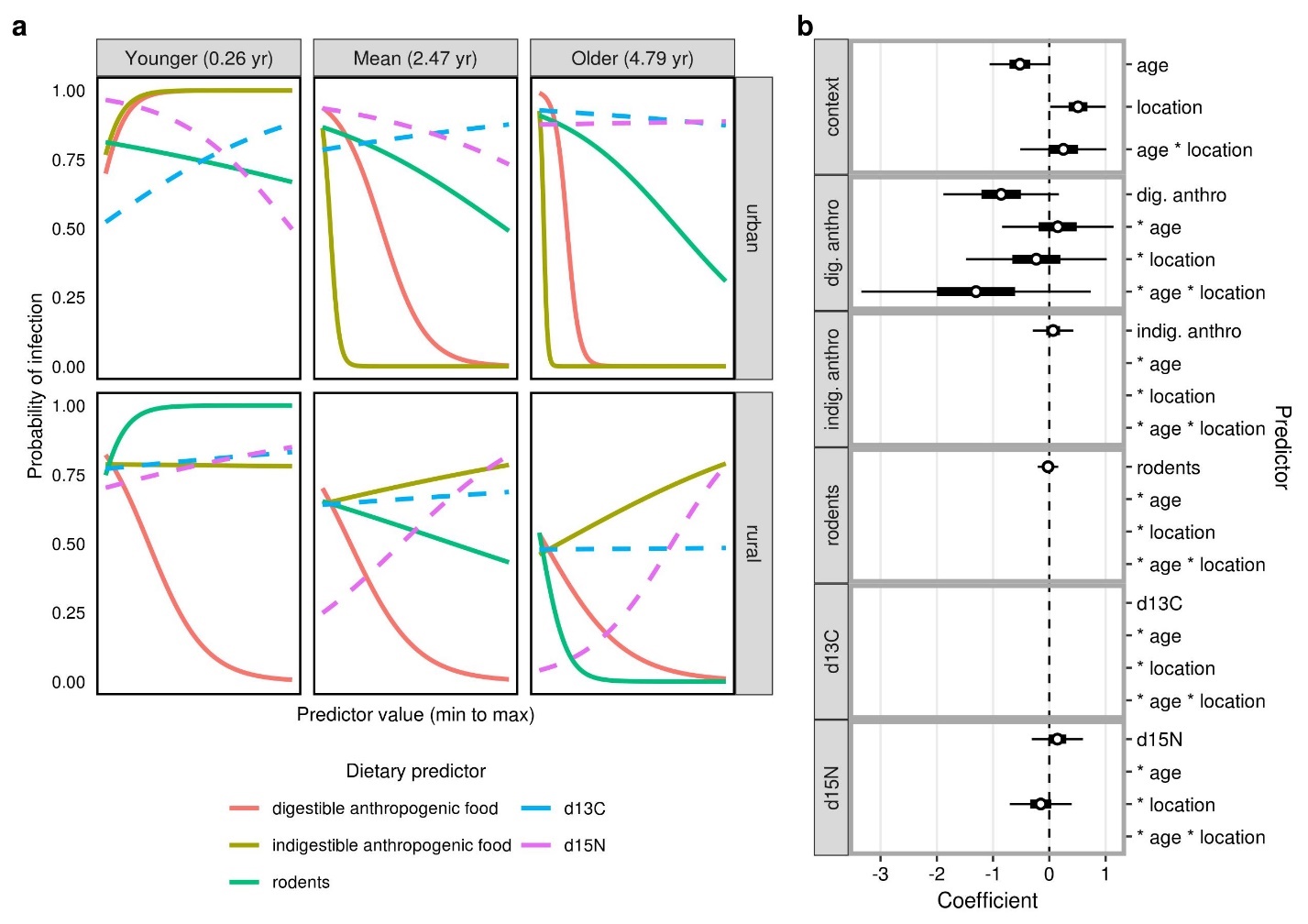


#### S4 Figure.

Figure 3 reproduced based on qPCR-positive infections instead of biologically active infections. See Fig. 3 for a complete legend.


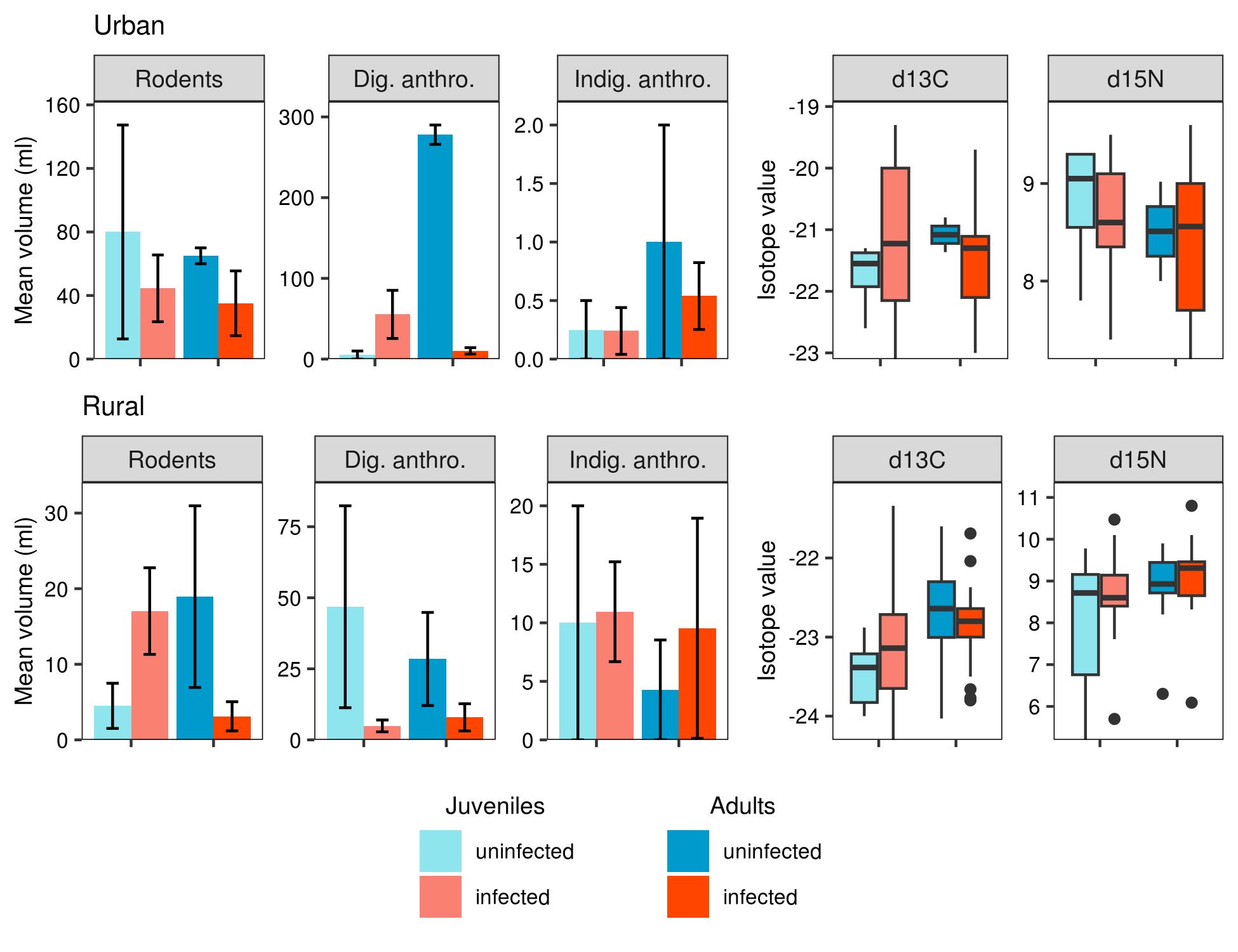


#### S5 Figure.

Figure 4 reproduced based on qPCR-positive infections instead of biologically active infections. See Fig. 4 for a complete legend.
