# Supplementary material for: Age-dependent relationships among diet, body condition, and Echinococcus multilocularis infection in urban coyotes
Source: PLoS One. 2023 Aug 30;18(8):e0290755. doi: 10.1371/journal.pone.0290755 (PMC10468061; doi:10.1371/journal.pone.0290755)
Supplement: S3 Appendix — Full numerical results for (i) statistical tests for differences in infection status or intensity as a function of contextual variables or diet and (ii) generalized linear models predicting infection status or intensity as a function of these same variables. These data support Figs 3, 4 and 6 in the main manuscript text. (DOCX) [file pone.0290755.s003.docx]

**Age-dependent relationships among diet, body condition, and *Echinococcus multilocularis* infection in urban coyotes**

S. Sugden, D.K. Steckler, D. Sanderson, B. Abercrombie, D. Abercrombie, M.A. Seguin, K. Ford, C.C. St. Clair

**S3 APPENDIX: Predictors of *E. multilocularis* infection**

Full numerical results for (i) statistical tests for differences in infection status or intensity as a function of contextual variables or diet and (ii) generalized linear models predicting infection status or intensity as a function of these same variables. These data support Figs. 3, 4, and 6 in the main manuscript text.

*Material begins on the following page.*

#### S1 Table.

Univariate tests for differences in infection status and intensity as a function of location, age, sex, condition, and diet. For infection status, relationships with categorical variables (location, sex) were assessed with chi-squared tests, and relationships with continuous variables (age, condition, and diet) were assessed using Wilcox rank sum tests. For infection intensity, relationships with categorical and numerical variables were assessed using Wilcox rank sum tests and Spearman’s correlation, respectively.

|  |  |  | **Status** | |  | **Intensity** | |
| --- | --- | --- | --- | --- | --- | --- | --- |
|  |  |  | Chi-squared | |  | Wilcox rank sum | |
| Variable | | | χ^2^ | p |  | W | p |
| Context | | |  |  |  |  |  |
|  | location | | 1.610 | 0.205 |  | 1260 | 0.204 |
|  | sex | | 0.124 | 0.725 |  | 1543 | 0.917 |
|  |  |  |  |  |  |  |  |
|  |  |  | Wilcox rank sum | |  | Spearman correlation | |
| Context | | | W | p |  | R | p |
|  | age | | 2137.5 | **0.001** |  | -0.342 | **0.000** |
|  | body condition | | 1998.0 | **0.012** |  | -0.254 | **0.007** |
|  | KFI | | 1780.0 | 0.214 |  | -0.160 | **0.093** |
|  | spleen size | | 1508.5 | 0.740 |  | -0.035 | 0.713 |
|  |  |  |  |  |  |  |  |
| Stomach contents | | |  |  |  |  |  |
|  | total food volume | | 1698.5 | 0.442 |  | -0.085 | 0.372 |
|  | diet diversity | | 1486.0 | 0.641 |  | 0.056 | 0.558 |
|  |  |  |  |  |  |  |  |
|  | anthropogenic food | | 1540.0 | 0.876 |  | -0.008 | 0.930 |
|  |  | digestible | 1729.0 | 0.299 |  | -0.091 | 0.342 |
|  |  | indigestible | 1298.5 | **0.063** |  | 0.159 | **0.095** |
|  | prey items | | 1752.5 | 0.276 |  | -0.098 | 0.303 |
|  |  | rodent | 1731.0 | 0.293 |  | -0.086 | 0.365 |
|  |  | meso-mammal | 1787.5 | 0.110 |  | -0.131 | 0.170 |
|  |  | ungulate | 1597.0 | 0.830 |  | -0.057 | 0.548 |
|  |  | bird | 1307.0 | **0.039** |  | 0.188 | **0.047** |
|  |  | insects | 1528.0 | 0.603 |  | 0.106 | 0.265 |
|  | vegetation | | 1375.5 | 0.245 |  | 0.046 | 0.634 |
|  | fruit | | 1471.5 | 0.289 |  | 0.103 | 0.279 |
|  |  |  |  |  |  |  |  |
| Stable isotopes | | |  |  |  |  |  |
|  | d13C | | 1643.5 | 0.654 |  | -0.021 | 0.826 |
|  | d15N | | 1650.5 | 0.625 |  | -0.065 | 0.498 |


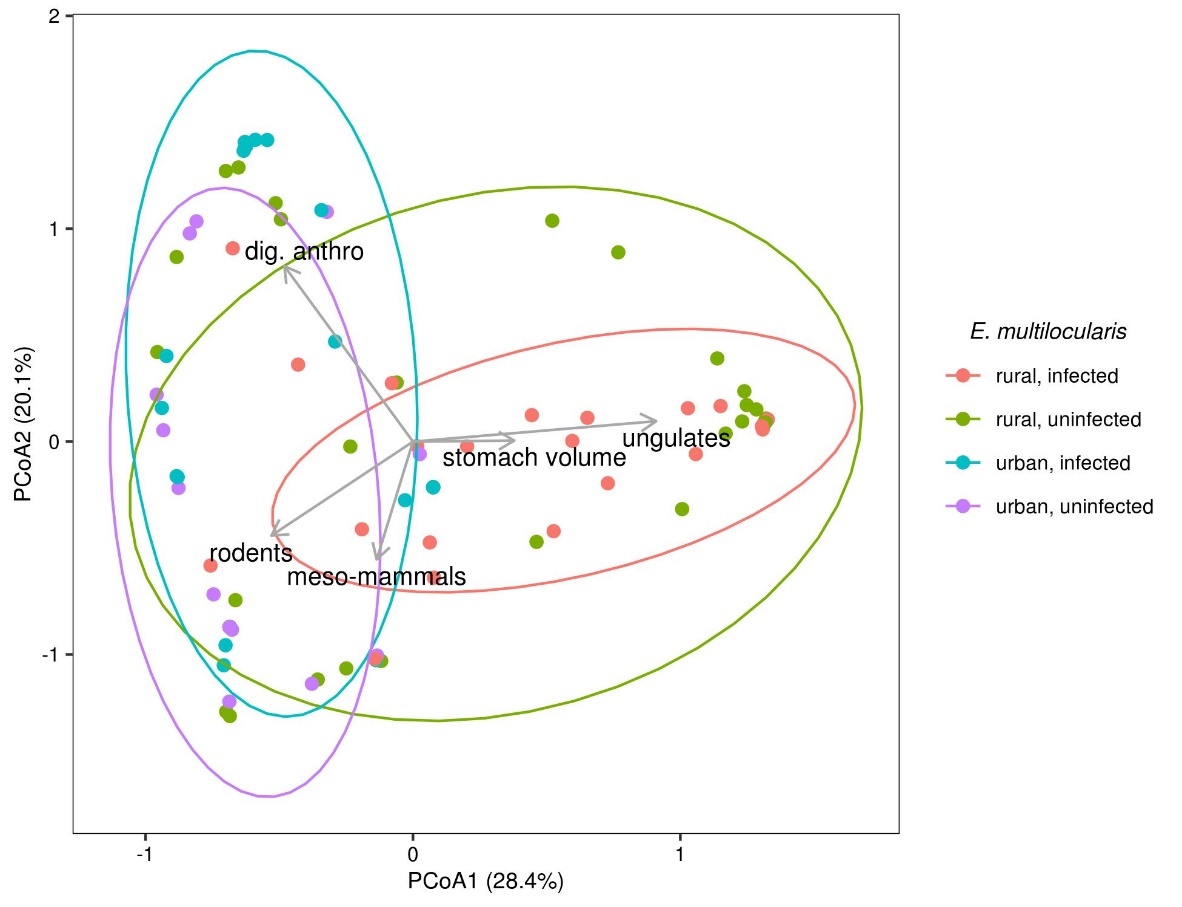


#### S1 Figure.

Ordination of stomach contents. Coyotes are colored by *E. multilocularis* infection status, and ellipses show the 90% confidence intervals for infection statuses (colored ellipses) and locations (dashed ellipses). Significant (p < 0.05) vectors are overlaid on the ordination. Overall, stomach contents are better indicators of location (urban vs. rural) than infection status. Results from PERMANOVA and vector-fitting analyses are provided in **Table S6**.

#### S2 Table.

Clustering and vector-fitting results from stomach content ordinations. We calculated the Bray-Curtis distance between each coyote stomach based on the relative abundances (by volume) of stomach contents. Significant clustering associations due to location, age, infection status, and infection intensity were assessed using a permutational multivariate analysis of variance (PERMANOVA). In addition, we overlayed stomach contents, age, and infection intensity on the resulting ordination and tested for significant relationships using the ‘envfit’ function in the R package *vegan*.

| PERMANOVA | | | | |
| --- | --- | --- | --- | --- |
| Predictor | R^2^ | F | df | p |
| location | 0.098 | 10.938 | 1 | 0.001 |
| infection status | 0.016 | 1.821 | 1 | 0.109 |
| age x location | 0.016 | 1.754 | 1 | 0.128 |
| age | 0.014 | 1.600 | 1 | 0.175 |
| infection intensity | 0.007 | 0.827 | 1 | 0.553 |
| residuals | 0.849 |  | 95 |  |
| total | 1 |  | 100 |  |
|  |  |  |  |  |
| Vector-fitting | | |  |  |
| Vector | R^2^ | p |  |  |
| dig. anthro | 0.910 | **0.001** |  |  |
| ungulates | 0.836 | **0.001** |  |  |
| rodents | 0.475 | **0.001** |  |  |
| meso-mammals | 0.325 | **0.001** |  |  |
| stomach volume | 0.144 | **0.001** |  |  |
| age | 0.054 | 0.073 |  |  |
| fruit | 0.043 | 0.083 |  |  |
| indig. anthro | 0.026 | 0.296 |  |  |
| vegetation | 0.008 | 0.670 |  |  |
| infection intensity | 0.004 | 0.840 |  |  |
| birds | 0.002 | 0.925 |  |  |

#### S3 Table.

Top-ranked models (ΔAIC_C_ < 2) and goodness-of-fit statistics for logistic regression models predicting infection status. For each model, coefficients were standardized by their partial standard deviation and are shown with their 95% confidence interval in parentheses. Regressions were evaluated using predictive accuracy and Cohen’s kappa in five-fold cross validation. McFadden’s R^2^ is also shown for all models. In addition to the top-ranked models for each response variable, the table shows results for the null model, global model, and the model based on age and location only.

|  | context | | |  |  | digestible anthropogenic food | | | |
| --- | --- | --- | --- | --- | --- | --- | --- | --- | --- |
| Model rank | age | location | age x location |  |  | univariate | x age | x location | x age  x location |
| 1 | -0.56 (0.58) | 0.45 (0.46) | 0.38 (0.39) |  |  | -0.70 (0.73) | -0.80 (0.83) | 0.59 (0.62) | 0.63 (0.65) |
| 2 | -0.52 (0.56) | 0.42 (0.46) | 0.34 (0.37) |  |  | -0.62 (0.67) | -0.76 (0.82) | 0.53 (0.57) | 0.59 (0.64) |
| 3 | -0.56 (0.58) | 0.46 (0.47) | 0.39 (0.40) |  |  | -0.77 (0.80) | -0.85 (0.89) | 0.65 (0.67) | 0.68 (0.70) |
| 4 | -0.46 (0.55) | 0.38 (0.45) | 0.30 (0.36) |  |  | -0.54 (0.64) | -0.64 (0.77) | 0.46 (0.54) | 0.49 (0.59) |
| 5 | -0.43 (0.55) | 0.35 (0.45) | 0.29 (0.37) |  |  | -0.52 (0.67) | -0.64 (0.82) | 0.44 (0.57) | 0.50 (0.64) |
| 6 | -0.57 (0.58) | 0.46 (0.47) | 0.39 (0.39) |  |  | -0.73 (0.74) | -0.84 (0.86) | 0.62 (0.63) | 0.66 (0.68) |
| 7 | -0.50 (0.57) | 0.41 (0.46) | 0.33 (0.37) |  |  | -0.65 (0.74) | -0.78 (0.89) | 0.55 (0.63) | 0.61 (0.70) |
| 8 | -0.38 (0.54) | 0.31 (0.44) | 0.25 (0.36) |  |  | -0.45 (0.64) | -0.54 (0.77) | 0.38 (0.54) | 0.41 (0.60) |
| 9 | -0.48 (0.56) | 0.39 (0.45) | 0.31 (0.36) |  |  | -0.60 (0.70) | -0.70 (0.82) | 0.51 (0.59) | 0.54 (0.64) |
| global | -0.54 (0.54) | 0.43 (0.44) | 0.36 (0.37) |  |  | -0.73 (0.74) | -0.84 (0.86) | 0.62 (0.62) | 0.67 (0.68) |
| age * loc | -0.71 (0.42) | 0.29 (0.33) | 0.45 (0.29) |  |  | - | - | - | - |
| null | - | - | - |  |  | - | - | - | - |
|  |  |  |  |  |  |  |  |  |  |
|  | indigestible anthropogenic food | | | |  | rodents | | | |
| Model rank | univariate | x age | x location | x age  x location |  | univariate | x age | x location | x age  x location |
| 1 | 0.90 (0.86) | 0.87 (0.87) | - | - |  | -0.44 (0.41) | -0.47 (0.38) | - | - |
| 2 | 0.92 (0.87) | 0.71 (0.89) | - | - |  | -0.42 (0.41) | -0.45 (0.38) | - | - |
| 3 | 0.94 (0.87) | 0.90 (0.88) | 0.28 (0.46) | - |  | -0.47 (0.41) | -0.51 (0.39) | - | - |
| 4 | 0.85 (0.87) | 0.70 (0.90) | - | - |  | -0.41 (0.40) | -0.44 (0.38) | - | - |
| 5 | 0.63 (0.57) | - | - | - |  | -0.39 (0.40) | -0.43 (0.38) | - | - |
| 6 | 0.92 (0.87) | 0.85 (0.87) | - | - |  | -0.44 (0.41) | -0.47 (0.39) | - | - |
| 7 | 0.87 (0.88) | 0.75 (0.90) | 0.30 (0.45) | - |  | -0.45 (0.41) | -0.49 (0.39) | - | - |
| 8 | 0.60 (0.56) | - | - | - |  | -0.38 (0.40) | -0.41 (0.37) | - | - |
| 9 | 0.89 (0.87) | 0.74 (0.90) | 0.28 (0.44) | - |  | -0.43 (0.41) | -0.47 (0.39) | - | - |
| global | 0.86 (0.83) | 0.67 (0.84) | 0.06 (0.43) | -0.14 (0.4) |  | -0.14 (0.39) | 0.00 (0.37) | -0.26 (0.36) | -0.31 (0.33) |
| age * loc | - | - | - | - |  | - | - | - | - |
| null | - | - | - | - |  | - | - | - | - |
|  |  |  |  |  |  |  |  |  |  |
|  |  |  |  |  |  |  |  |  |  |
|  |  |  |  |  |  |  |  |  |  |
|  |  |  |  |  |  |  |  |  |  |
|  | d13C | | | |  | d15N | | | |
| Model rank | univariate | x age | x location | x age  x location |  | univariate | x age | x location | x age  x location |
| 1 | -0.41 (0.42) | -0.55 (0.45) | - | - |  | 0.31 (0.47) | 0.24 (0.62) | -0.40 (0.38) | -0.53 (0.46) |
| 2 | -0.49 (0.43) | -0.47 (0.42) | 0.32 (0.39) | - |  | -0.20 (0.47) | -0.77 (0.60) | - | - |
| 3 | -0.39 (0.43) | -0.57 (0.45) | - | - |  | 0.31 (0.47) | 0.26 (0.63) | -0.41 (0.38) | -0.52 (0.47) |
| 4 | -0.37 (0.41) | -0.48 (0.40) | - | - |  | -0.26 (0.47) | -0.77 (0.60) | - | - |
| 5 | -0.49 (0.42) | -0.46 (0.43) | 0.32 (0.39) | - |  | -0.19 (0.48) | -0.87 (0.63) | - | - |
| 6 | -0.44 (0.43) | -0.51 (0.45) | 0.22 (0.40) | - |  | 0.30 (0.48) | 0.16 (0.63) | -0.38 (0.38) | -0.46 (0.47) |
| 7 | -0.48 (0.43) | -0.49 (0.43) | 0.34 (0.40) | - |  | -0.18 (0.47) | -0.66 (0.61) | - | - |
| 8 | -0.35 (0.40) | -0.46 (0.40) | - | - |  | -0.24 (0.48) | -0.87 (0.63) | - | - |
| 9 | -0.33 (0.41) | -0.49 (0.41) | - | - |  | -0.23 (0.47) | -0.66 (0.61) | - | - |
| global | -0.33 (0.40) | -0.20 (0.41) | 0.15 (0.38) | -0.03 (0.4) |  | 0.27 (0.44) | 0.15 (0.58) | -0.38 (0.36) | -0.43 (0.42) |
| age * loc | - | - | - | - |  | - | - | - | - |
| null | - | - | - | - |  | - | - | - | - |
|  |  |  |  |  |  |  |  |  |  |
|  | model fit statistics | | | |  |  |  |  |  |
| Model rank | ΔAIC_C_ | accuracy (sd) | Kappa | McFadden R^2^ |  |  |  |  |  |
| 1 | 0 | 68.7% (11.0) | 0.37 (0.22) | 0.385 |  |  |  |  |  |
| 2 | 1.00 | 69.5% (9.9) | 0.39 (0.20) | 0.367 |  |  |  |  |  |
| 3 | 1.03 | **73.1% (12.3)** | **0.46 (0.25)** | **0.394** |  |  |  |  |  |
| 4 | 1.18 | 67.8% (11.7) | 0.36 (0.23) | 0.353 |  |  |  |  |  |
| 5 | 1.48 | 67.7% (11.9) | 0.35 (0.24) | 0.352 |  |  |  |  |  |
| 6 | 1.62 | 69.6% (11.2) | 0.39 (0.22) | 0.391 |  |  |  |  |  |
| 7 | 1.67 | 71.3% (9.4) | 0.43 (0.19) | 0.378 |  |  |  |  |  |
| 8 | 1.71 | 65.1% (8.5) | 0.30 (0.17) | 0.338 |  |  |  |  |  |
| 9 | 1.84 | 69.5% (10.9) | 0.39 (0.22) | 0.363 |  |  |  |  |  |
| global | 12.77 | 70.4% (12.7) | 0.41 (0.25) | 0.412 |  |  |  |  |  |
| age * loc | 27.85 | 64.2% (8.6) | 0.29 (0.17) | 0.089 |  |  |  |  |  |
| null | 40.42 | - | - | - |  |  |  |  |  |

#### S4 Table.

Top-ranked models (ΔAIC_C_ < 2) and goodness-of-fit statistics for negative binomial models predicting natural log-transformed infection intensity. For each model, coefficients were standardized by their partial standard deviation and are shown with their 95% confidence interval in parentheses. Regressions were evaluated using root mean squared error (RMSE) and mean average error (MAE). McFadden’s R^2^ is also shown for all models. In addition to the top-ranked models for each response variable, the table shows results for the null model, global model, and the model based on age and location only.

|  | context | | |  |  | digestible anthropogenic food | | | |
| --- | --- | --- | --- | --- | --- | --- | --- | --- | --- |
| Model rank | age | location | age x location |  |  | univariate | x age | x location | x age  x location |
| 1 | -0.73 (0.27) | 0.51 (0.22) | 0.37 (0.18) |  |  | -0.28 (0.23) | - | 0.23 (0.20) | - |
| 2 | -0.79 (0.27) | 0.51 (0.22) | 0.35 (0.18) |  |  | -0.29 (0.23) | - | 0.24 (0.20) | - |
| 3 | -0.75 (0.27) | 0.44 (0.22) | 0.33 (0.18) |  |  | -0.29 (0.23) | - | 0.24 (0.20) | - |
| 4 | -0.83 (0.26) | 0.51 (0.22) | 0.38 (0.17) |  |  | -0.28 (0.23) | - | 0.24 (0.20) | - |
| 5 | -0.74 (0.27) | 0.51 (0.22) | 0.36 (0.18) |  |  | -0.28 (0.24) | - | 0.24 (0.20) | - |
| 6 | -0.18 (0.26) | 0.16 (0.22) | 0.11 (0.17) |  |  | -0.15 (0.24) | -0.14 (0.23) | 0.13 (0.20) | 0.11 (0.17) |
| 7 | -0.17 (0.26) | 0.16 (0.22) | 0.11 (0.18) |  |  | -0.15 (0.24) | -0.14 (0.23) | 0.13 (0.20) | 0.11 (0.17) |
| 8 | -0.73 (0.26) | 0.49 (0.22) | 0.08 (0.17) |  |  | -0.28 (0.23) | - | 0.23 (0.20) | - |
| 9 | -0.53 (0.26) | 0.42 (0.22) | 0.13 (0.17) |  |  | -0.27 (0.24) | - | 0.23 (0.20) | - |
| 10 | -0.73 (0.26) | 0.51 (0.22) | 0.36 (0.17) |  |  | -0.27 (0.23) | - | 0.23 (0.20) | - |
| 11 | -0.77 (0.26) | 0.45 (0.22) | 0.35 (0.17) |  |  | -0.28 (0.23) | - | 0.24 (0.20) | - |
| 12 | -0.52 (0.26) | 0.43 (0.22) | 0.18 (0.17) |  |  | -0.26 (0.23) | - | 0.22 (0.20) | - |
| 13 | -0.17 (0.26) | 0.16 (0.22) | 0.11 (0.18) |  |  | -0.15 (0.24) | -0.14 (0.23) | 0.13 (0.20) | 0.10 (0.17) |
| global | -0.17 (0.22) | 0.14 (0.19) | 0.10 (0.14) |  |  | -0.15 (0.20) | -0.14 (0.19) | 0.12 (0.17) | 0.10 (0.14) |
| age * loc | -0.58 (0.30) | 0.27 (0.25) | 0.31 (0.21) |  |  | - | - | - | - |
| null | - | - | - |  |  | - | - | - | - |
|  |  |  |  |  |  |  |  |  |  |
|  | indigestible anthropogenic food | | | |  | rodents | | | |
| Model rank | univariate | x age | x location | x age  x location |  | univariate | x age | x location | x age  x location |
| 1 | 0.21 (0.25) | 0.41 (0.23) | 0.36 (0.22) | - |  | -0.42 (0.22) | -0.41 (0.20) | - | - |
| 2 | 0.14 (0.25) | 0.34 (0.23) | 0.37 (0.22) | - |  | -0.41 (0.22) | -0.41 (0.20) | - | - |
| 3 | 0.12 (0.25) | 0.31 (0.23) | 0.31 (0.22) | - |  | -0.39 (0.22) | -0.38 (0.20) | - | - |
| 4 | 0.15 (0.24) | 0.36 (0.22) | 0.38 (0.22) | - |  | -0.44 (0.22) | -0.43 (0.20) | - | - |
| 5 | 0.23 (0.25) | 0.38 (0.23) | 0.35 (0.22) | - |  | -0.28 (0.23) | -0.44 (0.21) | -0.1 (0.21) | - |
| 6 | 0.2 (0.25) | 0.42 (0.23) | 0.36 (0.22) | - |  | -0.33 (0.22) | -0.32 (0.20) | - | - |
| 7 | 0.12 (0.25) | 0.34 (0.23) | 0.32 (0.22) | - |  | -0.3 (0.22) | -0.29 (0.20) | - | - |
| 8 | 0.21 (0.25) | 0.41 (0.23) | 0.33 (0.22) | -0.09 (0.17) |  | -0.42 (0.22) | -0.42 (0.20) | - | - |
| 9 | 0.09 (0.24) | 0.27 (0.22) | 0.38 (0.22) | - |  | -0.08 (0.23) | -0.08 (0.20) | -0.26 (0.21) | -0.21 (0.19) |
| 10 | 0.21 (0.25) | 0.41 (0.23) | 0.36 (0.22) | - |  | -0.42 (0.22) | -0.41 (0.20) | - | - |
| 11 | 0.13 (0.25) | 0.33 (0.23) | 0.32 (0.22) | - |  | -0.4 (0.22) | -0.39 (0.20) | - | - |
| 12 | 0.15 (0.24) | 0.32 (0.22) | 0.36 (0.22) | - |  | -0.09 (0.23) | -0.08 (0.20) | -0.23 (0.21) | -0.19 (0.19) |
| 13 | 0.14 (0.25) | 0.37 (0.23) | 0.37 (0.22) | - |  | -0.32 (0.22) | -0.32 (0.20) | - | - |
| global | 0.12 (0.20) | 0.31 (0.18) | 0.10 (0.19) | -0.19 (0.14) |  | -0.10 (0.19) | -0.01 (0.17) | -0.23 (0.17) | -0.24 (0.15) |
| age * loc | - | - | - | - |  | - | - | - | - |
| null | - | - | - | - |  | - | - | - | - |
|  |  |  |  |  |  |  |  |  |  |
|  | d13C | | | |  | d15N | | | |
| Model rank | univariate | x age | x location | x age  x location |  | univariate | x age | x location | x age  x location |
| 1 | -0.28 (0.20) | -0.23 (0.18) | 0.32 (0.19) | 0.17 (0.17) |  | - | - | - | - |
| 2 | -0.17 (0.20) | -0.17 (0.19) | 0.25 (0.19) | - |  | - | - | - | - |
| 3 | -0.12 (0.20) | - | 0.22 (0.19) | - |  | - | - | - | - |
| 4 | -0.18 (0.19) | -0.18 (0.18) | 0.25 (0.18) | - |  | 0.24 (0.24) | - | -0.17 (0.18) | - |
| 5 | -0.26 (0.20) | -0.22 (0.18) | 0.3 (0.19) | 0.17 (0.17) |  | - | - | - | - |
| 6 | -0.24 (0.20) | -0.2 (0.18) | 0.29 (0.19) | 0.16 (0.17) |  | - | - | - | - |
| 7 | -0.1 (0.20) | - | 0.19 (0.19) | - |  | - | - | - | - |
| 8 | -0.28 (0.20) | -0.23 (0.18) | 0.33 (0.19) | 0.18 (0.16) |  | - | - | - | - |
| 9 | -0.15 (0.19) | -0.18 (0.18) | 0.23 (0.18) | - |  | - | - | - | - |
| 10 | -0.26 (0.20) | -0.21 (0.18) | 0.31 (0.19) | 0.16 (0.17) |  | 0.12 (0.23) | - | - | - |
| 11 | -0.12 (0.20) | - | 0.22 (0.19) | - |  | 0.18 (0.24) | - | - | - |
| 12 | -0.24 (0.19) | -0.2 (0.18) | 0.28 (0.18) | 0.14 (0.16) |  | - | - | - | - |
| 13 | -0.15 (0.20) | -0.16 (0.18) | 0.23 (0.19) | - |  | - | - | - | - |
| global | -0.16 (0.16) | -0.13 (0.15) | 0.18 (0.15) | 0.06 (0.14) |  | 0.12 (0.20) | 0.07 (0.23) | -0.17 (0.15) | -0.13 (0.14) |
| age * loc | - | - | - | - |  | - | - | - | - |
| null | - | - | - | - |  | - | - | - | - |
|  |  |  |  |  |  |  |  |  |  |
|  | model statistics | | | |  |  |  |  |  |
| Model rank | ΔAIC_C_ | RMSE (sd) | MAE (sd) | McFadden R^2^ |  |  |  |  |  |
| 1 | 0 | 3.78 (2.78) | 2.5 (1.44) | 0.123 |  |  |  |  |  |
| 2 | 0.93 | 2.92 (0.91) | 2.19 (0.79) | 0.117 |  |  |  |  |  |
| 3 | 1.38 | 3.18 (1.15) | 2.31 (0.82) | 0.111 |  |  |  |  |  |
| 4 | 1.68 | 2.86 (0.89) | 2.14 (0.64) | 0.125 |  |  |  |  |  |
| 5 | 1.68 | 3.87 (2.34) | 2.59 (1.3) | 0.125 |  |  |  |  |  |
| 6 | 1.71 | 337.56 (745.14) | 75.47 (162.67) | 0.130 |  |  |  |  |  |
| 7 | 1.76 | 83.11 (177.7) | 19.88 (38.84) | 0.120 |  |  |  |  |  |
| 8 | 1.76 | 4.03 (3.12) | 2.63 (1.56) | 0.125 |  |  |  |  |  |
| 9 | 1.81 | 3.1 (0.84) | 2.27 (0.71) | 0.125 |  |  |  |  |  |
| 10 | 1.87 | 3.68 (2.42) | 2.54 (1.34) | 0.125 |  |  |  |  |  |
| 11 | 1.89 | 3.35 (1.64) | 2.42 (0.97) | 0.115 |  |  |  |  |  |
| 12 | 1.92 | 3.43 (1.52) | 2.45 (1.02) | 0.130 |  |  |  |  |  |
| 13 | 1.93 | 98.76 (212.91) | 23.24 (46.51) | 0.125 |  |  |  |  |  |
| global | 8.69 | 3.12 (0.84) | 2.37 (0.22) | 0.156 |  |  |  |  |  |
| age * loc | 26.39 | 2.88 (0.25) | 2.21 (0.65) | 0.028 |  |  |  |  |  |
| null | 36.03 | - | - | - |  |  |  |  |  |


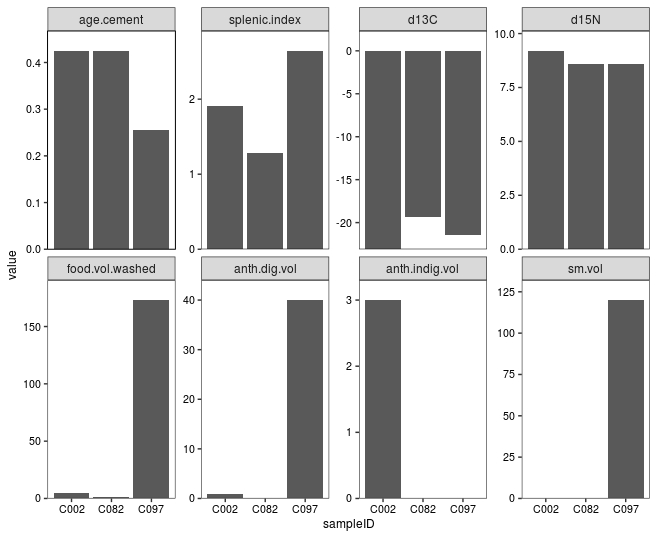


#### S2 Figure.

Age and dietary characteristics of the three coyotes with >10,000 *Echinococcus* scolexes in their intestines. All three coyotes with urban. The coyotes (identified C002, C082, and C097) harbored 30 000, 55 000, and 24 000 worms, respectively.

*To confirm that weighting urban samples more heavily in our models (to account for uneven sample sizes) did not substantially affect the model outcomes, we compared results between weighted and unweighted models, as shown in Tables S4 and S5.*

#### S4 Table.

Results from all univariate and interaction models in which urban samples were weighted proportionally to their representation in our sample pool. Each focal variable was run four models: (*i*) as a univariate predictors and again in interactions with (*ii*) age, (*iii*) location, and (*iv*) the two-way interaction between age and location. Chi-squared tests were used to compare each model to the equivalent model in which the focal variable did not appear.

|  | **likelihood ratio tests (univariate)** | | | | | | | | |
| --- | --- | --- | --- | --- | --- | --- | --- | --- | --- |
|  | **prevalence** | | | |  | **intensity** | | | |
| **Variable** | **AIC** | **χ^2^** | **df** | **p** |  | **AIC** | **χ^2^** | **df** | **p** |
| Context |  |  |  |  |  |  |  |  |  |
| location | 213.74 | 2.37 | 1 | 0.124 |  | 571.67 | 1.28 | 1 | 0.257 |
| age | 210.86 | 5.25 | 1 | 0.022 |  | 567.54 | 5.41 | 1 | 0.020 |
| sex | 215.94 | 0.17 | 1 | 0.681 |  | 572.90 | 0.05 | 1 | 0.830 |
| body condition | 209.43 | 6.68 | 1 | 0.010 |  | 567.26 | 5.69 | 1 | 0.017 |
| KFI | 214.80 | 1.30 | 1 | 0.253 |  | 571.58 | 1.37 | 1 | 0.242 |
| spleen size | 216.03 | 0.08 | 1 | 0.782 |  | 571.32 | 1.63 | 1 | 0.202 |
|  |  |  |  |  |  |  |  |  |  |
| Stomach contents |  |  |  |  |  |  |  |  |  |
| total food volume | 215.77 | 0.34 | 1 | 0.560 |  | 572.95 | 0.00 | 1 | 0.992 |
| diet diversity | 216.06 | 0.05 | 1 | 0.831 |  | 572.23 | 0.72 | 1 | 0.397 |
|  |  |  |  |  |  |  |  |  |  |
| anthropogenic food | 216.09 | 0.02 | 1 | 0.893 |  | 572.52 | 0.43 | 1 | 0.514 |
| digestible | 215.76 | 0.35 | 1 | 0.555 |  | 572.39 | 0.56 | 1 | 0.456 |
| indigestible | 214.38 | 1.73 | 1 | 0.189 |  | 572.95 | 0.00 | 1 | 0.990 |
| prey items | 215.88 | 0.22 | 1 | 0.635 |  | 572.92 | 0.03 | 1 | 0.867 |
| rodent | 215.96 | 0.14 | 1 | 0.704 |  | 572.93 | 0.02 | 1 | 0.898 |
| meso-mammal | 216.08 | 0.03 | 1 | 0.867 |  | 572.78 | 0.17 | 1 | 0.679 |
| ungulate | 215.85 | 0.26 | 1 | 0.612 |  | 572.95 | 0.00 | 1 | 0.985 |
| bird | 215.64 | 0.47 | 1 | 0.492 |  | 572.63 | 0.32 | 1 | 0.574 |
| insects | 210.81 | 5.30 | 1 | 0.021 |  | 569.93 | 3.02 | 1 | 0.082 |
| vegetation | 216.08 | 0.03 | 1 | 0.868 |  | 571.92 | 1.03 | 1 | 0.311 |
| fruit | 215.98 | 0.13 | 1 | 0.720 |  | 572.93 | 0.02 | 1 | 0.880 |
|  |  |  |  |  |  |  |  |  |  |
| Stable isotopes |  |  |  |  |  |  |  |  |  |
| d13C | 215.46 | 0.65 | 1 | 0.421 |  | 572.02 | 0.93 | 1 | 0.336 |
| d15N | 216.06 | 0.05 | 1 | 0.820 |  | 572.62 | 0.33 | 1 | 0.565 |
|  |  |  |  |  |  |  |  |  |  |
|  |  |  |  |  |  |  |  |  |  |
|  | **likelihood ratio tests - age * focal variable vs. age only** | | | | | | | | |
|  | **prevalence** | | | |  | **intensity** | | | |
| **Variable** | **AIC** | **χ^2^** | **df** | **p** |  | **AIC** | **χ^2^** | **df** | **p** |
| Context |  |  |  |  |  |  |  |  |  |
| location | 201.20 | 13.66 | 2 | 0.001 |  | 560.81 | 10.73 | 2 | 0.005 |
| age | - | - | - | - |  | - | - | - | - |
| sex | 212.96 | 1.89 | 2 | 0.388 |  | 568.61 | 2.93 | 2 | 0.231 |
| body condition | 210.49 | 4.37 | 2 | 0.113 |  | 565.55 | 5.99 | 2 | 0.050 |
| KFI | 207.92 | 6.94 | 2 | 0.031 |  | 560.31 | 11.23 | 2 | 0.004 |
| spleen size | 200.41 | 14.45 | 2 | 0.001 |  | 560.14 | 11.40 | 2 | 0.003 |
|  |  |  |  |  |  |  |  |  |  |
| Stomach contents |  |  |  |  |  |  |  |  |  |
| total food volume | 210.00 | 4.86 | 2 | 0.088 |  | 568.25 | 3.29 | 2 | 0.193 |
| diet diversity | 214.76 | 0.10 | 2 | 0.953 |  | 570.81 | 0.73 | 2 | 0.693 |
|  |  |  |  |  |  |  |  |  |  |
| anthropogenic food | 212.05 | 2.81 | 2 | 0.245 |  | 570.67 | 0.86 | 2 | 0.649 |
| digestible | 207.62 | 7.23 | 2 | 0.027 |  | 564.66 | 6.87 | 2 | 0.032 |
| indigestible | 213.13 | 1.73 | 2 | 0.422 |  | 569.47 | 2.06 | 2 | 0.356 |
| prey items | 211.58 | 3.28 | 2 | 0.194 |  | 568.32 | 3.22 | 2 | 0.200 |
| rodent | 204.07 | 10.79 | 2 | 0.005 |  | 562.32 | 9.22 | 2 | 0.010 |
| meso-mammal | 213.93 | 0.93 | 2 | 0.628 |  | 571.04 | 0.50 | 2 | 0.778 |
| ungulate | 211.10 | 3.76 | 2 | 0.152 |  | 567.02 | 4.52 | 2 | 0.105 |
| bird | 209.31 | 5.55 | 2 | 0.062 |  | 568.79 | 2.74 | 2 | 0.254 |
| insects | 200.51 | 14.34 | 2 | 0.001 |  | 569.54 | 2.00 | 2 | 0.368 |
| vegetation | 213.98 | 0.88 | 2 | 0.644 |  | 570.13 | 1.41 | 2 | 0.495 |
| fruit | 211.41 | 3.45 | 2 | 0.179 |  | 568.58 | 2.96 | 2 | 0.227 |
|  |  |  |  |  |  |  |  |  |  |
| Stable isotopes |  |  |  |  |  |  |  |  |  |
| d13C | 210.43 | 4.42 | 2 | 0.109 |  | 562.57 | 8.97 | 2 | 0.011 |
| d15N | 210.99 | 3.87 | 2 | 0.145 |  | 566.88 | 4.66 | 2 | 0.097 |
|  |  |  |  |  |  |  |  |  |  |
|  | **likelihood ratio tests - loc * focal variable vs. location only** | | | | | | | | |
|  | **prevalence** | | | |  | **intensity** | | | |
| **Variable** | **AIC** | **χ^2^** | **df** | **p** |  | **AIC** | **χ^2^** | **df** | **p** |
| Context |  |  |  |  |  |  |  |  |  |
| location | - | - | - | - |  | - | - | - | - |
| age | 201.198 | 16.54 | 2 | 0.000 |  | 560.807 | 14.86 | 2 | 0.001 |
| sex | 217.56 | 0.17 | 2 | 0.917 |  | 575.41 | 0.25 | 2 | 0.882 |
| body condition | 212.21 | 5.53 | 2 | 0.063 |  | 570.42 | 5.24 | 2 | 0.073 |
| KFI | 217.36 | 0.38 | 2 | 0.829 |  | 574.46 | 1.21 | 2 | 0.547 |
| spleen size | 215.93 | 1.81 | 2 | 0.405 |  | 570.63 | 5.03 | 2 | 0.081 |
|  |  |  |  |  |  |  |  |  |  |
| Stomach contents |  |  |  |  |  |  |  |  |  |
| total food volume | 217.365 | 0.37 | 2 | 0.831 |  | 575.077 | 0.59 | 2 | 0.745 |
| diet diversity | 217.368 | 0.37 | 2 | 0.832 |  | 573.509 | 2.16 | 2 | 0.340 |
|  |  |  |  |  |  |  |  |  |  |
| anthropogenic food | 216.72 | 1.02 | 2 | 0.602 |  | 573.29 | 2.38 | 2 | 0.304 |
| digestible | 209.53 | 8.21 | 2 | 0.017 |  | 563.57 | 12.10 | 2 | 0.002 |
| indigestible | 213.739 | 4.00 | 2 | 0.136 |  | 574.122 | 1.54 | 2 | 0.462 |
| prey items | 217.14 | 0.60 | 2 | 0.742 |  | 575.09 | 0.58 | 2 | 0.749 |
| rodent | 217.10 | 0.64 | 2 | 0.728 |  | 575.49 | 0.18 | 2 | 0.915 |
| meso-mammal | 217.44 | 0.30 | 2 | 0.861 |  | 574.91 | 0.76 | 2 | 0.685 |
| ungulate | 217.50 | 0.23 | 2 | 0.890 |  | 575.18 | 0.49 | 2 | 0.784 |
| bird | 215.40 | 2.33 | 2 | 0.312 |  | 574.99 | 0.68 | 2 | 0.712 |
| insects | 210.18 | 7.56 | 2 | 0.023 |  | 570.21 | 5.46 | 2 | 0.065 |
| vegetation | 217.66 | 0.07 | 2 | 0.964 |  | 574.56 | 1.11 | 2 | 0.574 |
| fruit | 216.05 | 1.68 | 2 | 0.431 |  | 574.33 | 1.33 | 2 | 0.513 |
|  |  |  |  |  |  |  |  |  |  |
| Stable isotopes |  |  |  |  |  |  |  |  |  |
| d13C | 212.84 | 4.89 | 2 | 0.087 |  | 573.01 | 2.65 | 2 | 0.265 |
| d15N | 217.55 | 0.19 | 2 | 0.910 |  | 574.71 | 0.95 | 2 | 0.621 |
|  |  |  |  |  |  |  |  |  |  |
|  | **likelihood ratio tests - age * loc * focal variable vs. age * loc only** | | | | | | | | |
|  | **prevalence** | | | |  | **intensity** | | | |
| **Variable** | **AIC** | **χ^2^** | **df** | **p** |  | **AIC** | **χ^2^** | **df** | **p** |
| Context |  |  |  |  |  |  |  |  |  |
| location | - | - | - | - |  | - | - | - | - |
| age | - | - | - | - |  | - | - | - | - |
| sex | 206.99 | 2.2096 | 4 | 0.697 |  | 566.46 | 2.35 | 4 | 0.672 |
| body condition | 204.40 | 4.7978 | 4 | 0.309 |  | 562.65 | 6.15 | 4 | 0.188 |
| KFI | 208.30 | 0.901 | 4 | 0.924 |  | 565.04 | 3.76 | 4 | 0.439 |
| spleen size | 198.83 | 10.371 | 4 | 0.035 |  | 551.82 | 16.98 | 4 | 0.002 |
|  |  |  |  |  |  |  |  |  |  |
| Stomach contents |  |  |  |  |  |  |  |  |  |
| total food volume | 207.12 | 2.08 | 4 | 0.722 |  | 566.918 | 1.89 | 4 | 0.756 |
| diet diversity | 206.99 | 2.21 | 4 | 0.697 |  | 561.92 | 6.87 | 4 | 0.142 |
|  |  |  |  |  |  |  |  |  |  |
| anthropogenic food | 201.01 | 8.19 | 4 | 0.085 |  | 555.46 | 13.35 | 4 | 0.010 |
| digestible | 192.65 | 16.54 | 4 | 0.002 |  | 551.49 | 17.32 | 4 | 0.002 |
| indigestible | 204.42 | 4.78 | 4 | 0.311 |  | 557.474 | 11.33 | 4 | 0.023 |
| prey items | 207.47 | 1.73 | 4 | 0.785 |  | 566.28 | 2.53 | 4 | 0.640 |
| rodent | 193.83 | 15.37 | 4 | 0.004 |  | 550.10 | 18.70 | 4 | 0.001 |
| meso-mammal | 199.05 | 10.15 | 4 | 0.038 |  | 563.07 | 5.74 | 4 | 0.220 |
| ungulate | 203.10 | 6.10 | 4 | 0.192 |  | 563.92 | 4.89 | 4 | 0.299 |
| bird | 203.55 | 5.65 | 4 | 0.227 |  | 562.69 | 6.12 | 4 | 0.191 |
| insects | 193.07 | 16.13 | 4 | 0.003 |  | 562.17 | 6.64 | 4 | 0.156 |
| vegetation | 204.39 | 4.80 | 4 | 0.308 |  | 566.59 | 2.21 | 4 | 0.697 |
| fruit | 203.28 | 5.92 | 4 | 0.205 |  | 563.53 | 5.28 | 4 | 0.260 |
|  |  |  |  |  |  |  |  |  |  |
| Stable isotopes |  |  |  |  |  |  |  |  |  |
| d13C | 206.91 | 2.29 | 4 | 0.682 |  | 566.78 | 2.03 | 4 | 0.730 |
| d15N | 203.08 | 6.11 | 4 | 0.191 |  | 565.12 | 3.68 | 4 | 0.451 |

#### S5 Table.

Results from all univariate and interaction models in which urban samples were not weighted. Each focal variable was run four models: (*i*) as a univariate predictors and again in interactions with (*ii*) age, (*iii*) location, and (*iv*) the two-way interaction between age and location. Chi-squared tests were used to compare each model to the equivalent model in which the focal variable did not appear.

|  | **likelihood ratio tests (univariate)** | | | | | | | | |  |
| --- | --- | --- | --- | --- | --- | --- | --- | --- | --- | --- |
|  | **prevalence** | | | |  | **intensity** | | | |  |
| **Variable** | **AIC** | **χ^2^** | **df** | **p** |  | **AIC** | **χ^2^** | **df** | **p** |  |
| Context |  |  |  |  |  |  |  |  |  |  |
| location | 157.51 | 1.612 | 1 | 0.204 |  | 440.63 | 0.911 | 1 | 0.340 |  |
| age | 150.70 | 8.420 | 1 | 0.004 |  | 434.99 | 6.594 | 1 | 0.010 |  |
| sex | 159.00 | 0.124 | 1 | 0.725 |  | 441.38 | 0.080 | 1 | 0.777 |  |
| body condition | 153.99 | 5.128 | 1 | 0.024 |  | 437.98 | 3.616 | 1 | 0.057 |  |
| KFI | 158.08 | 1.046 | 1 | 0.306 |  | 440.71 | 0.773 | 1 | 0.379 |  |
| spleen size | 159.12 | 0.002 | 1 | 0.967 |  | 440.74 | 0.705 | 1 | 0.401 |  |
|  |  |  |  |  |  |  |  |  |  |  |
| Stomach contents |  |  |  |  |  |  |  |  |  |  |
| total food volume | 158.96 | 0.157 | 1 | 0.691 |  | 441.43 | 0.019 | 1 | 0.891 |  |
| diet diversity | 159.00 | 0.118 | 1 | 0.731 |  | 441.26 | 0.218 | 1 | 0.641 |  |
|  |  |  |  |  |  |  |  |  |  |  |
| anthropogenic food | 158.99 | 0.127 | 1 | 0.721 |  | 440.95 | 0.508 | 1 | 0.476 |  |
| digestible | 158.32 | 0.805 | 1 | 0.370 |  | 440.80 | 0.658 | 1 | 0.417 |  |
| indigestible | 157.09 | 2.032 | 1 | 0.154 |  | 441.45 | 0.001 | 1 | 0.977 |  |
| prey items | 159.05 | 0.073 | 1 | 0.787 |  | 441.37 | 0.076 | 1 | 0.783 |  |
| rodent | 159.06 | 0.060 | 1 | 0.806 |  | 441.44 | 0.005 | 1 | 0.943 |  |
| meso-mammal | 159.04 | 0.086 | 1 | 0.770 |  | 441.17 | 0.264 | 1 | 0.608 |  |
| ungulate | 158.98 | 0.141 | 1 | 0.708 |  | 441.44 | 0.007 | 1 | 0.931 |  |
| bird | 158.48 | 0.645 | 1 | 0.422 |  | 441.09 | 0.349 | 1 | 0.555 |  |
| insects | 156.59 | 2.528 | 1 | 0.112 |  | 439.92 | 1.731 | 1 | 0.188 |  |
| vegetation | 159.12 | 0.005 | 1 | 0.945 |  | 440.55 | 0.920 | 1 | 0.337 |  |
| fruit | 158.82 | 0.301 | 1 | 0.583 |  | 441.43 | 0.020 | 1 | 0.886 |  |
|  |  |  |  |  |  |  |  |  |  |  |
| Stable isotopes |  |  |  |  |  |  |  |  |  |  |
| d13C | 159.05 | 0.073 | 1 | 0.787 |  | 441.15 | 0.335 | 1 | 0.563 |  |
| d15N | 159.12 | 0.004 | 1 | 0.952 |  | 441.35 | 0.093 | 1 | 0.760 |  |
|  |  |  |  |  |  |  |  |  |  |  |
|  |  |  |  |  |  |  |  |  |  |  |
|  | **likelihood ratio tests - age * focal variable vs. age only** | | | | | | | | |  |
|  | **prevalence** | | | |  | **intensity** | | | |  |
| **Variable** | **AIC** | **χ^2^** | **df** | **p** |  | **AIC** | **χ^2^** | **df** | **p** |  |
| Context |  |  |  |  |  |  |  |  |  |  |
| location | 145.14 | 9.56 | 2 | 0.008 |  | 430.23 | 8.75 | 2 | 0.013 |  |
| age | - | - | - | - |  | - | - | - | - |  |
| sex | 153.37 | 1.33 | 2 | 0.514 |  | 436.52 | 2.46 | 2 | 0.292 |  |
| body condition | 152.85 | 1.85 | 2 | 0.397 |  | 434.71 | 4.28 | 2 | 0.118 |  |
| KFI | 149.41 | 5.30 | 2 | 0.071 |  | 429.60 | 9.39 | 2 | 0.009 |  |
| spleen size | 145.21 | 9.49 | 2 | 0.009 |  | 429.63 | 9.36 | 2 | 0.009 |  |
|  |  |  |  |  |  |  |  |  |  |  |
| Stomach contents |  |  |  |  |  |  |  |  |  |  |
| total food volume | 151.54 | 3.16 | 2 | 0.206 |  | 436.22 | 2.77 | 2 | 0.251 |  |
| diet diversity | 154.64 | 0.06 | 2 | 0.969 |  | 438.26 | 0.73 | 2 | 0.696 |  |
|  |  |  |  |  |  |  |  |  |  |  |
| anthropogenic food | 153.91 | 0.79 | 2 | 0.673 |  | 438.37 | 0.62 | 2 | 0.734 |  |
| digestible | 150.25 | 4.45 | 2 | 0.108 |  | 433.51 | 5.47 | 2 | 0.065 |  |
| indigestible | 152.67 | 2.04 | 2 | 0.361 |  | 436.44 | 2.55 | 2 | 0.280 |  |
| prey items | 152.07 | 2.64 | 2 | 0.268 |  | 435.92 | 3.07 | 2 | 0.216 |  |
| rodent | 149.96 | 4.74 | 2 | 0.093 |  | 434.85 | 4.13 | 2 | 0.127 |  |
| meso-mammal | 154.35 | 0.35 | 2 | 0.841 |  | 438.51 | 0.47 | 2 | 0.789 |  |
| ungulate | 152.14 | 2.56 | 2 | 0.278 |  | 435.15 | 3.83 | 2 | 0.147 |  |
| bird | 151.22 | 3.48 | 2 | 0.176 |  | 437.26 | 1.73 | 2 | 0.421 |  |
| insects | 146.90 | 7.80 | 2 | 0.020 |  | 437.97 | 1.02 | 2 | 0.602 |  |
| vegetation | 154.01 | 0.69 | 2 | 0.708 |  | 438.12 | 0.86 | 2 | 0.650 |  |
| fruit | 151.98 | 2.72 | 2 | 0.257 |  | 436.66 | 2.33 | 2 | 0.312 |  |
|  |  |  |  |  |  |  |  |  |  |  |
| Stable isotopes |  |  |  |  |  |  |  |  |  |  |
| d13C | 151.11 | 3.59 | 2 | 0.166 |  | 431.69 | 7.30 | 2 | 0.026 |  |
| d15N | 152.87 | 1.83 | 2 | 0.401 |  | 435.92 | 3.07 | 2 | 0.216 |  |
|  |  |  |  |  |  |  |  |  |  |  |
|  |  |  |  |  |  |  |  |  |  |  |
|  | **likelihood ratio tests - loc * focal variable vs. location only** | | | | | | | | |  |
|  | **prevalence** | | | |  | **intensity** | | | |  |
| **Variable** | **AIC** | **χ^2^** | **df** | **p** |  | **AIC** | **χ^2^** | **df** | **p** |  |
| Context |  |  |  |  |  |  |  |  |  |  |
| location | - | - | - | - |  | - | - | - | - |  |
| age | 145.14 | 16.37 | 2 | 0.000 |  | 430.23 | 14.40 | 2 | 0.007 |  |
| sex | 161.38 | 0.13 | 2 | 0.938 |  | 444.40 | 0.24 | 2 | 0.888 |  |
| body condition | 157.17 | 4.34 | 2 | 0.114 |  | 441.45 | 3.18 | 2 | 0.203 |  |
| KFI | 161.14 | 0.37 | 2 | 0.829 |  | 443.95 | 0.68 | 2 | 0.712 |  |
| spleen size | 160.60 | 0.91 | 2 | 0.635 |  | 441.91 | 2.73 | 2 | 0.256 |  |
|  |  |  |  |  |  |  |  |  |  |  |
| Stomach contents |  |  |  |  |  |  |  |  |  |  |
| total food volume | 161.32 | 0.19 | 2 | 0.910 |  | 444.2471 | 0.39 | 2 | 0.824 |  |
| diet diversity | 161.15 | 0.36 | 2 | 0.836 |  | 443.543 | 1.09 | 2 | 0.580 |  |
|  |  |  |  |  |  |  |  |  |  |  |
| anthropogenic food | 160.51 | 1.00 | 2 | 0.608 |  | 442.39 | 2.24 | 2 | 0.326 |  |
| digestible | 153.30 | 8.21 | 2 | 0.017 |  | 432.94 | 11.70 | 2 | 0.003 |  |
| indigestible | 158.08 | 3.43 | 2 | 0.180 |  | 443.8207 | 0.81 | 2 | 0.666 |  |
| prey items | 161.21 | 0.30 | 2 | 0.860 |  | 444.18 | 0.45 | 2 | 0.798 |  |
| rodent | 161.12 | 0.39 | 2 | 0.821 |  | 444.51 | 0.12 | 2 | 0.941 |  |
| meso-mammal | 161.24 | 0.27 | 2 | 0.873 |  | 443.98 | 0.66 | 2 | 0.721 |  |
| ungulate | 161.39 | 0.12 | 2 | 0.942 |  | 444.34 | 0.29 | 2 | 0.863 |  |
| bird | 159.87 | 1.64 | 2 | 0.440 |  | 444.02 | 0.62 | 2 | 0.735 |  |
| insects | 156.56 | 4.95 | 2 | 0.084 |  | 440.55 | 4.09 | 2 | 0.130 |  |
| vegetation | 161.47 | 0.04 | 2 | 0.980 |  | 443.67 | 0.97 | 2 | 0.616 |  |
| fruit | 160.38 | 1.13 | 2 | 0.567 |  | 443.63 | 1.00 | 2 | 0.607 |  |
|  |  |  |  |  |  |  |  |  |  |  |
| Stable isotopes |  |  |  |  |  |  |  |  |  |  |
| d13C | 156.95 | 4.56 | 2 | 0.103 |  | 442.51 | 2.12 | 2 | 0.346 |  |
| d15N | 161.38 | 0.13 | 2 | 0.938 |  | 444.04 | 0.59 | 2 | 0.743 |  |
|  |  |  |  |  |  |  |  |  |  |  |
|  |  |  |  |  |  |  |  |  |  |  |
|  | **likelihood ratio tests - age * loc * focal variable vs. age * loc only** | | | | | | | | | ests - age * loc * focal variable vs. age * loc only |
|  | **prevalence** | | | |  | **intensity** | | | |  |
| **Variable** | **AIC** | **χ^2^** | **df** | **p** |  | **AIC** | **χ^2^** | **df** | **p** |  |
| Context |  |  |  |  |  |  |  |  |  |  |
| location | - | - | - | - |  | - | - | - | - |  |
| age | - | - | - | - |  | - | - | - | - |  |
| sex | 151.33 | 1.82 | 4 | 0.770 |  | 436.14 | 2.10 | 4 | 0.718 |  |
| body condition | 150.24 | 2.90 | 4 | 0.575 |  | 434.36 | 3.88 | 4 | 0.423 |  |
| KFI | 152.29 | 0.85 | 4 | 0.931 |  | 435.17 | 3.07 | 4 | 0.547 |  |
| spleen size | 146.85 | 6.29 | 4 | 0.178 |  | 425.70 | 12.54 | 4 | 0.014 |  |
|  |  |  |  |  |  |  |  |  |  |  |
| Stomach contents |  |  |  |  |  |  |  |  |  |  |
| total food volume | 151.79 | 1.35 | 4 | 0.853 |  | 436.7509 | 1.48 | 4 | 0.830 |  |
| diet diversity | 151.77 | 1.37 | 4 | 0.849 |  | 432.5006 | 5.73 | 4 | 0.220 |  |
|  |  |  |  |  |  |  |  |  |  |  |
| anthropogenic food | 147.79 | 5.35 | 4 | 0.253 |  | 426.09 | 12.14 | 4 | 0.016 |  |
| digestible | 139.63 | 13.51 | 4 | 0.009 |  | 422.37 | 15.87 | 4 | 0.003 |  |
| indigestible | 148.93 | 4.21 | 4 | 0.378 |  | 427.7922 | 10.44 | 4 | 0.034 |  |
| prey items | 151.70 | 1.44 | 4 | 0.837 |  | 435.87 | 2.36 | 4 | 0.669 |  |
| rodent | 145.25 | 7.89 | 4 | 0.096 |  | 427.73 | 10.50 | 4 | 0.033 |  |
| meso-mammal | 147.13 | 6.01 | 4 | 0.198 |  | 434.01 | 4.22 | 4 | 0.377 |  |
| ungulate | 149.75 | 3.39 | 4 | 0.495 |  | 434.83 | 3.40 | 4 | 0.493 |  |
| bird | 149.55 | 3.59 | 4 | 0.464 |  | 434.45 | 3.78 | 4 | 0.436 |  |
| insects | 143.95 | 9.19 | 4 | 0.057 |  | 433.76 | 4.48 | 4 | 0.345 |  |
| vegetation | 150.27 | 2.88 | 4 | 0.579 |  | 436.82 | 1.42 | 4 | 0.841 |  |
| fruit | 148.24 | 4.90 | 4 | 0.298 |  | 433.74 | 4.49 | 4 | 0.344 |  |
|  |  |  |  |  |  |  |  |  |  |  |
| Stable isotopes |  |  |  |  |  |  |  |  |  |  |
| d13C | 151.71 | 1.43 | 4 | 0.839 |  | 436.91 | 1.32 | 4 | 0.858 |  |
| d15N | 149.44 | 3.71 | 4 | 0.447 |  | 435.64 | 2.59 | 4 | 0.628 |  |

**
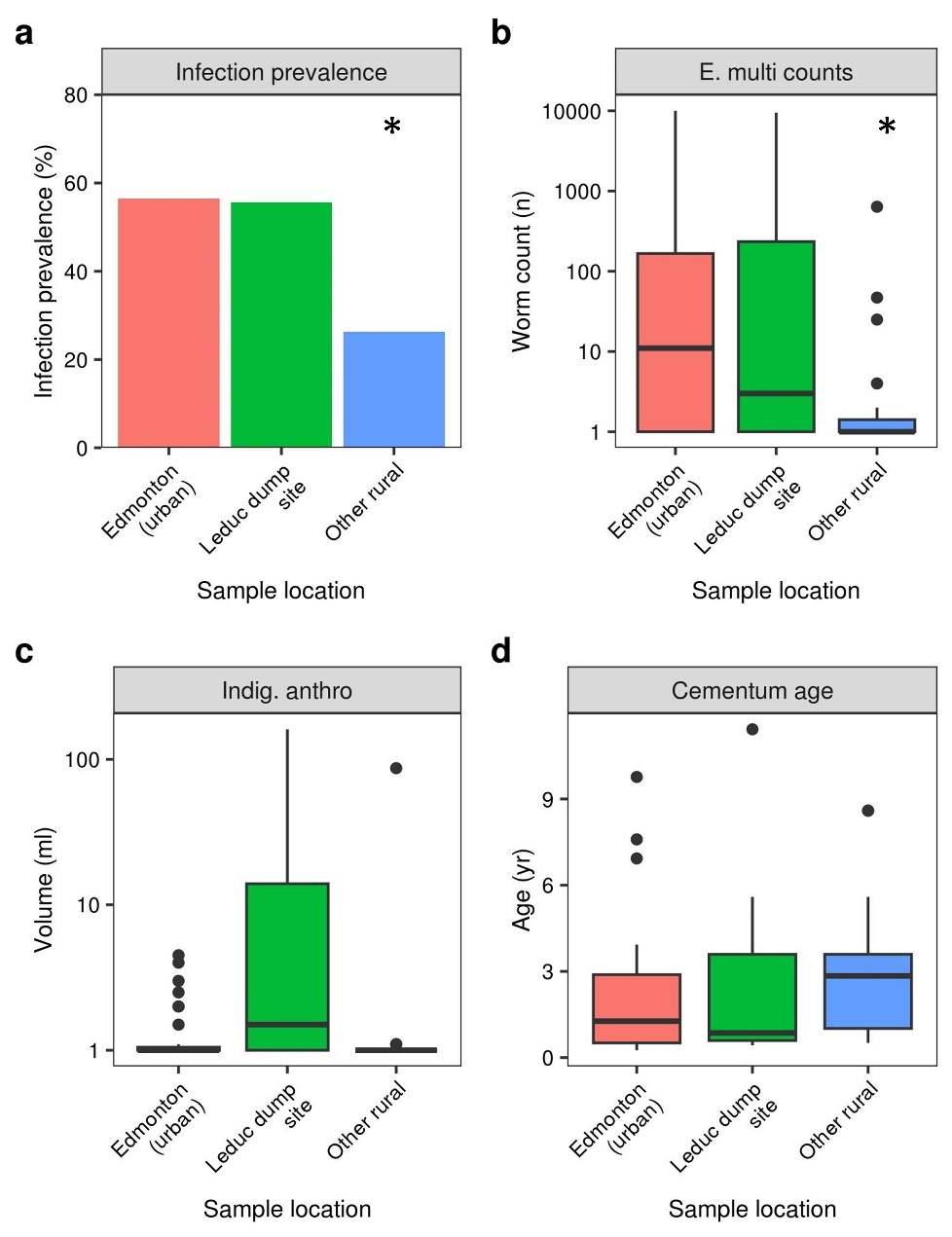
**

#### S3 Figure.

(**a**) Infection status, (**b**) intestinal worm counts, (**c**) indigestible anthropogenic food consumption, and (**d**) age distribution for urban coyotes compared to rural coyotes captured near the Leduc regional landfill and rural coyotes collected elsewhere. In general, coyotes captured close to the Leduc regional landfill were younger, consumed more indigestible anthropogenic food, and were more likely to be infected with *E. multilocularis* than rural coyotes collected elsewhere. Note that the total number of coyotes represented in this figure is n=85, as some rural coyotes were provided without capture locations. Worm counts were truncated at 10,000. Asterisks indicate significant (p < 0.05) differences between groups, as assessed using an ANOVA followed by Tukey’s *post hoc* test.
